# Dynamic expression patterns of *Irx3* and *Irx5* during germline nest breakdown and primordial follicle formation promote follicle survival in mouse ovaries

**DOI:** 10.1371/journal.pgen.1007488

**Published:** 2018-08-02

**Authors:** Anqi Fu, Sydney M. Oberholtzer, Stefan Bagheri-Fam, Raphael H. Rastetter, Claire Holdreith, Valeria L. Caceres, Steven V. John, Sarah A. Shaw, Kathleen J. Krentz, Xiaoyun Zhang, Chi-chung Hui, Dagmar Wilhelm, Joan S. Jorgensen

**Affiliations:** 1 Department of Comparative Biosciences, School of Veterinary Medicine, University of Wisconsin – Madison, Madison, Wisconsin, United States of America; 2 Department of Anatomy and Neuroscience, The University of Melbourne, Parkville, Victoria, Australia; 3 Department of Anatomy and Developmental Biology, Monash University, Clayton, Victoria, Australia; 4 Genome Editing and Animal Models Core, Biotechnology Center, University of Wisconsin – Madison, Madison, Wisconsin, United States of America; 5 Program in Developmental & Stem Cell Biology, The Hospital for Sick Children and Department of Molecular Genetics, University of Toronto, Toronto, Ontario, Canada; Cornell University, UNITED STATES

## Abstract

Women and other mammalian females are born with a finite supply of oocytes that determine their reproductive lifespan. During fetal development, individual oocytes are enclosed by a protective layer of granulosa cells to form primordial follicles that will grow, mature, and eventually release the oocyte for potential fertilization. Despite the knowledge that follicles are dysfunctional and will die without granulosa cell-oocyte interactions, the mechanisms by which these cells establish communication is unknown. We previously identified that two members of the Iroquois homeobox transcription factor gene family, *Irx3* and *Irx5*, are expressed within developing ovaries but not testes. Deletion of both factors (*Irx3*^*-*^*Irx5*^*EGFP*^/*Irx3*^*-*^*Irx5*^*EGFP*^) disrupted granulosa cell-oocyte contact during early follicle development leading to oocyte death. Thus, we hypothesized that *Irx3* and *Irx5* are required to develop cell-cell communication networks to maintain follicle integrity and female fertility. A series of *Irx3* and *Irx5* mutant mouse models were generated to assess roles for each factor. While both *Irx3* and *Irx5* single mutant females were subfertile, their breeding outcomes and ovary histology indicated distinct causes. Careful analysis of *Irx3*- and *Irx5*-reporter mice linked the cause of this disparity to dynamic spatio-temporal changes in their expression patterns. Both factors marked the progenitor pre-granulosa cell population in fetal ovaries. At the critical phase of germline nest breakdown and primordial follicle formation however, *Irx3* and *Irx5* transitioned to oocyte- and granulosa cell-specific expression respectively. Further investigation into the cause of follicle death in *Irx3*^*-*^*Irx5*^*EGFP*^/*Irx3*^*-*^*Irx5*^*EGFP*^ ovaries uncovered specific defects in both granulosa cells and oocytes. Granulosa cell defects included poor contributions to basement membrane deposition and mis-localization of gap junction proteins. Granulosa cells and oocytes both presented fewer cell projections resulting in compromised cell-cell communication. Altogether, we conclude that *Irx3* and *Irx5* first work together to define the pregranulosa cell population of germline nests. During primordial follicle formation, they transition to oocyte- and granulosa cell-specific expression patterns where they cooperate in neighboring cells to build the foundation for follicle integrity. This foundation is left as their legacy of the essential oocyte-granulosa cell communication network that ensures and ultimately optimizes the integrity of the ovarian reserve and therefore, the female reproductive lifespan.

## Author summary

Fertility is a concern in women’s health, especially when the reported average age of mothers is rising (2016, CDC DB232). Of concern in aging mothers is ovarian follicle health, which requires active communication between its cell components that include a single egg and its surrounding granulosa support cells. The process used to establish cell-cell communication within follicles is unknown but begins during fetal development. We discovered that two related genes, *Irx3* and *Irx5*, direct network construction. If *Irx3* and *Irx5* are eliminated in mice, oocyte-granulosa cell contact is lost, and all follicles die within short order. Analysis of additional *Irx3* and *Irx5* mutant mice highlighted that each factor is important for establishing follicle integrity. During fetal development, they work together in the same cell to establish the identity of future granulosa support cells. Then, as the follicles form, they continue to work cooperatively, but in different cells. While *Irx5* expression remains in granulosa cells, *Irx3* expression transitions to the oocyte and together, they promote cell patterns and interacting zones to synchronize the follicle as an interactive unit. Although the work of *Irx3* and *Irx5* is finished in the perinatal ovary, their impact lasts a lifetime as it establishes long-term follicle integrity and therefore, fertility.

## Introduction

Mammalian neonatal ovaries are endowed with a finite and non-replenishable reserve of oocytes that will ultimately define the length of the female’s entire reproductive lifespan. The oocytes that define the ovarian reserve are maintained within primordial follicles, which are comprised of a single oocyte surrounded by a protective layer of somatic cells called granulosa cells. Once females reach reproductive age, some primordial follicles are recruited at regular intervals for maturation and potential ovulation or programmed atresia, but many of them will remain dormant for years. The integrity of those follicles depends on appropriate communication between oocytes and granulosa cells to ensure survival. Early depletion of primordial follicles and other follicle loss result in conditions such as premature ovarian insufficiency (POI), which poses great risk to a woman’s fertility and overall systemic health, but the underlying causes remain largely unknown [1, 2].

After sex determination in female (XX) gonads, primordial germ cells proliferate to form germline nests [3–5]. Just before birth in mice, germline nests break down to form primordial follicles through processes that include pre-granulosa cells extending cytoplasmic projections between oocytes and selective oocyte apoptosis [6–8]. Factors identified that promote this process include FOXL2, NOBOX, Notch and steroid hormone signaling pathways [9–13]. Later, when primordial follicles mature to the primary follicle stage, oocyte-derived signaling molecules including BMP15 and GDF9 begin to direct communication with surrounding granulosa cells to promote follicle growth and maturation [13–18]. As follicles mature, granulosa cells respond to autocrine, paracrine, and hormonal signals via IGF, KIT ligand, and gonadotropins, among others, to coordinate follicle development and eventually ovulation [15, 19–22]. The communication between oocyte and surrounding granulosa cells is key to each of these events and intercellular crosstalk begins as early as the germline nest and then oversees progression through primordial follicle formation, follicle maturation, and ovulation. The events that establish the initial interactions between pre-granulosa cells and oocyte within primordial and primary follicles, however, are still unknown.

Previously, we discovered that deletion of the *Fused toes* (*Ft*) locus, which includes *Irx3* and *Irx5*, caused POI with abnormal primordial follicle formation that failed to progress beyond the primary follicle stage [23]. IRX3 and IRX5, like all members of the Iroquois homeobox gene family, are characterized by an 11-amino acid *Iro* motif and a highly conserved DNA binding homeodomain that includes three extra amino acids, thereby distinguishing them as TALE (three amino acid loop extension) homeodomain transcription factors. Iroquois factors are known for their role in patterning and embryogenesis and are conserved from worms to vertebrates [24]. Among the six members of the Iroquois homeobox gene family, *Irx3*, *Irx5*, and *Irx6* comprise the *IrxB* cluster that resides on chromosomes 8 and 16 in mouse and human, respectively [25, 26]. *Irx3* and *Irx5* exhibit considerable transcript and protein homology and their expression patterns overlap in several developing tissues including the central nervous system, heart, gastrointestinal tract, skin, mammary gland, and limb [24–27]. Recently, our laboratory showed that both *Irx3* and *Irx5* were detected within the gonad in a sexually dimorphic pattern with enriched expression in developing ovaries, while *Irx6* was not detected [23, 27–31]. The timing of the peak of *Irx3* and *Irx5* expression corresponded to germline nest breakdown and primordial follicle formation, suggesting an important role in ovarian development [23]. This premise was supported by our findings that, like the *Ft* mutant mouse ovaries, targeted deletion of both *Irx3* and *Irx5* caused disruption of granulosa cell-oocyte contacts during early follicle development leading to oocyte death [3]. We therefore hypothesized that each Iroquois factor (IRX3 and IRX5) is critical to maintain follicle integrity and promote fertility. Here we report that deletion of either *Irx3* or *Irx5* impairs female fertility, but with distinct outcomes from each strain suggesting specific roles for each gene. This was supported by their dynamic expression profiles that are initially shared in pre-granulosa cells of germline nests and then subsequently diverge to granulosa cell- or oocyte-specific as nests breakdown into primordial follicles. Investigations into potential causes of follicle death in *Irx3* and *Irx5* double knockout ovaries uncovered defective cell biology in both granulosa cells and oocytes including abnormal deposition of the basement membrane, mis-localized gap junction proteins, and diminished extension processes emanating from both cell types. Taken together, we conclude that *Irx3* and *Irx5* work together in the same cells during fetal development, and then cooperate in neighboring cells to synchronize the formation of oocyte-granulosa cell interactions and promote granulosa cell function to establish the foundation required for follicle integrity and optimal female fertility.

## Results

### *Irx3* and *Irx5* are important for female fertility

Previously, we reported that follicles from *Fused Toes* (*Ft*) mutant mouse ovaries failed to mature as a result of defective granulosa cell-oocyte interactions. Of the six genes eliminated in the *Ft* mutation, only *Irx3* and *Irx5* exhibited an ovary-enriched expression profile suggesting that they played an integral role in follicle formation and maturation [23]. To test their roles in ovary development independent of the *Ft* mutation, we analyzed a series of *Irx3* and *Irx5* mutant mouse models.

**Fig 1:**
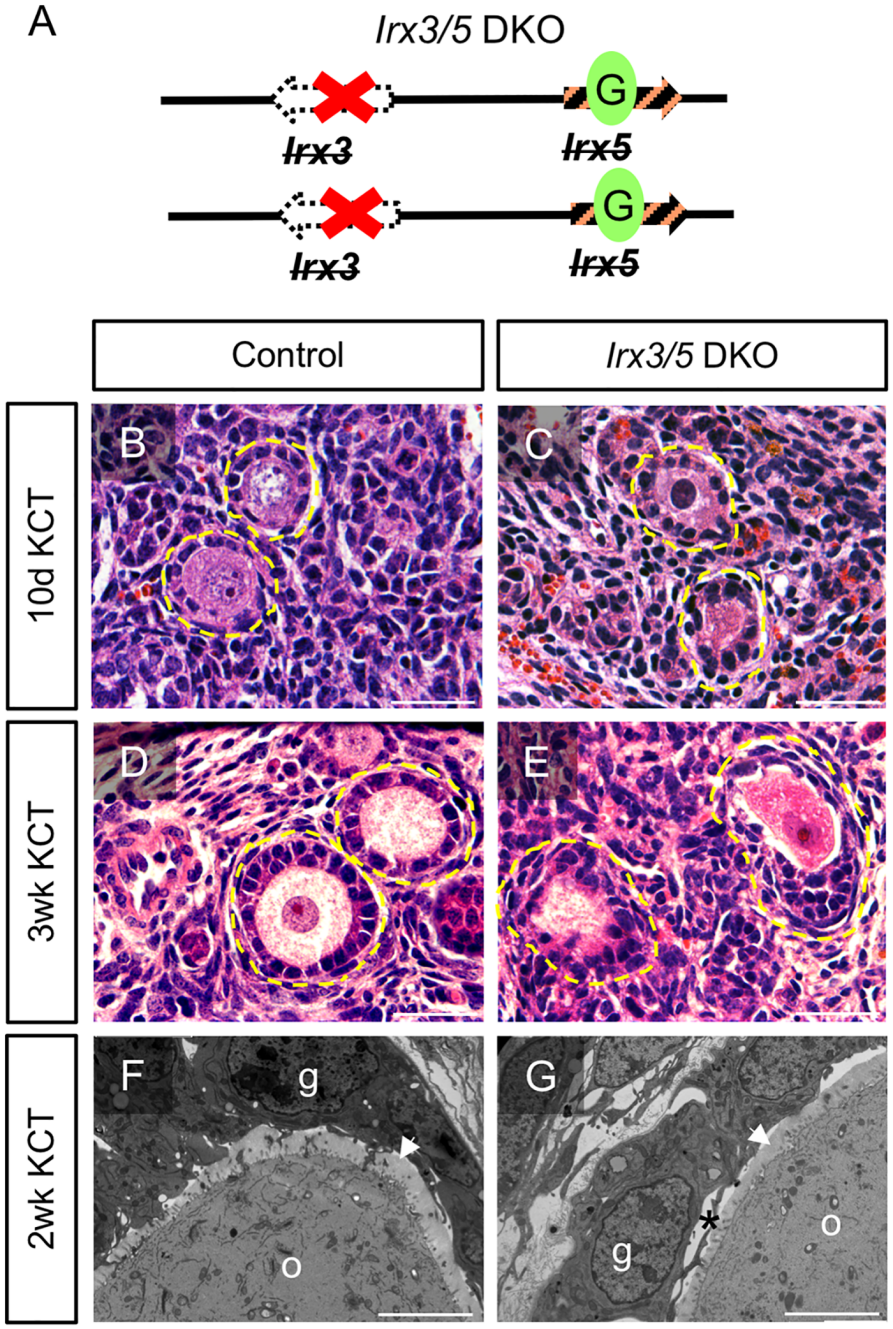
*Irx3/5* DKO causes abnormal granulosa cell morphology and disrupts granulosa cell-oocyte interactions. (A) Schematic drawing of the *Irx3* ^*-*^*Irx5*^*EGFP*^/*Irx3* ^*-*^*Irx5*^*EGFP*^ mutation (*Irx3/5* double knock out, *Irx3/5* DKO): *Irx3* is an engineered null mutation on both alleles and *Irx5* is rendered non-functional by the insertion of EGFP sequences (green oval labeled with a G) on both alleles. (B-E) H&E histology of control (B, D) and *Irx3/5* DKO (C, E) ovary grafts harvested 10 days (B, C) and 3 weeks (D, E) post kidney capsule transplant (KCT) surgery. Yellow dashed circle: outline of follicle. Scale bars: 50 μm. (F, G) Transmission electron micrographs of control (F) and *Irx3/5* DKO (G) ovary grafts harvested 2 weeks post KCT surgery. Asterisk (*): gap between oocyte and granulosa cells; g: granulosa cell; o: oocyte; white arrowhead: zona pellucida. Scale bars: 5 μm.

Similar to the *Ft* mutation, the *Irx3*^*-*^*Irx5*^*EGFP*^/*Irx3*^*-*^*Irx5*^*EGFP*^ double knockout (*Irx3/5* DKO, Fig 1A) is lethal in mice by approximately embryonic day 13.5–14.5 (E13.5–14.5) [32]. Ovaries from E12.5 wild type and *Irx3/5* DKO female embryos were transplanted under the kidney capsule (KCT) of ovariectomized nude mouse hosts for 10 days, 2 or 3 weeks to evaluate ovary development beyond the lethal stage. Ten days after surgery, both wild type control and mutant grafts contained follicles, suggesting that *Irx3* and *Irx5* are not necessary for the initiation of follicle formation (Fig 1B and 1C). Wild type primary follicles exhibited granulosa cells with their characteristic cuboidal shape surrounding and intimately associated with the oocyte in transplant grafts after 10 days and three weeks KCT (Fig 1B and 1D). In contrast, granulosa cells in primary follicles of *Irx3/5* DKO grafts were rounded in shape and unevenly distributed around an oocyte with disrupted cell-cell contacts (Fig 1C and 1E). Closer examination by transmission electron microscopy (TEM) verified significant gaps in granulosa cell-oocyte contacts in *Irx3/5* DKO follicles compared to the wild type control (Fig 1F and 1G)[3]. The relative incidence of abnormal follicle morphology was quantified. Abnormal follicles were defined as those that exhibited at least one of the following characteristics: mis-shaped granulosa cells (rounded, not cuboidal), non-uniform or wisp-like oocyte cytoplasm, presence of apoptotic bodies, asymmetric accumulation of granulosa cells relative to a central oocyte, and increased distances between granulosa cells and/or between granulosa cells and oocytes (S1 Fig). Compared to wild type (n = 44 follicles) grafts, *Irx3/5* DKO (n = 52 follicles) grafts harbored substantially higher percentages of abnormal primordial (14% vs 0%), primary (80% vs 17%) and secondary/pre-antral (90% vs 59%) follicles.

**Fig 2:**
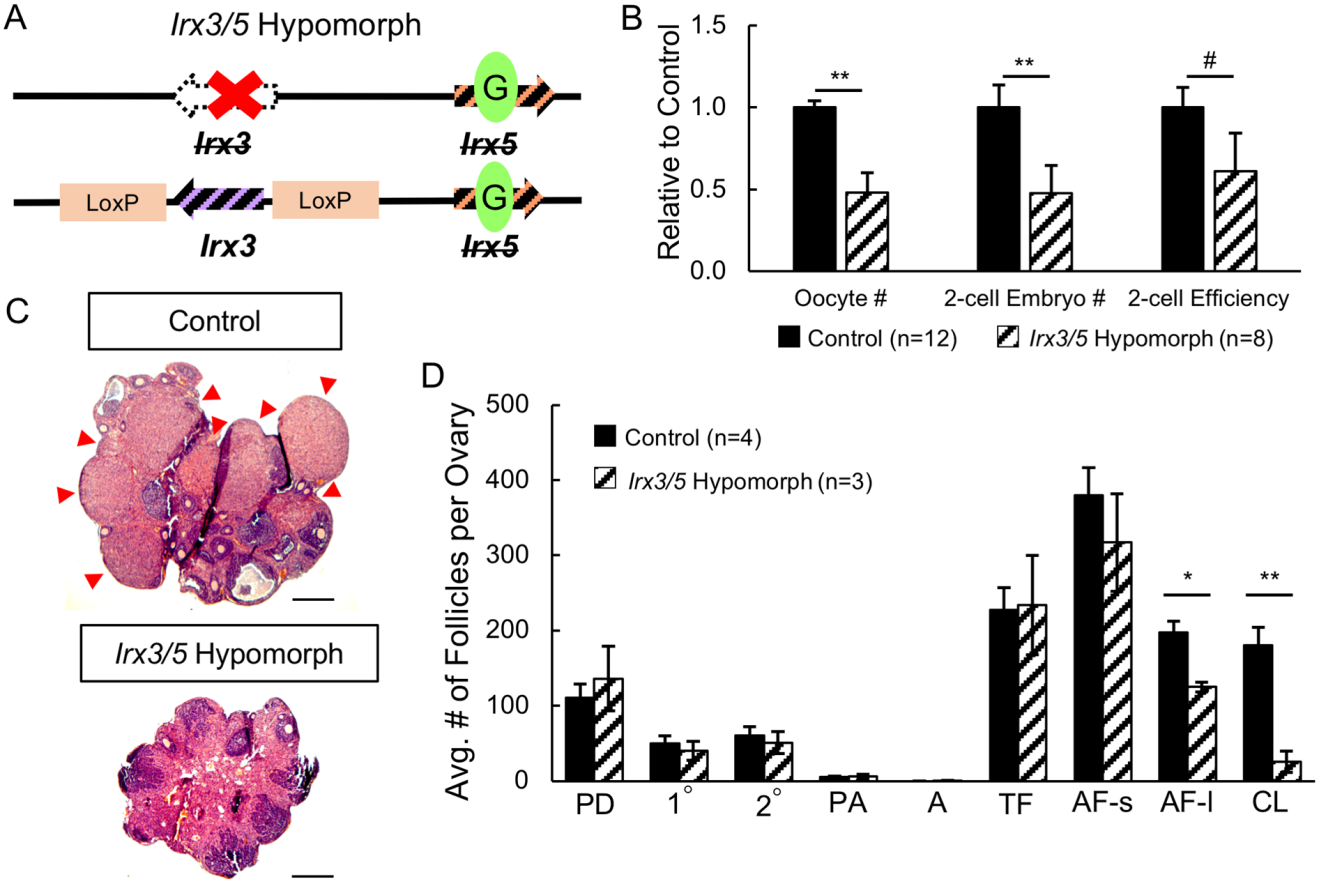
*Irx3/5* hypomorph mice fail to respond to ovulation signals. (A) Schematic drawing of the *Irx3* ^*-*^*Irx5*^*EGFP*^/*Irx3* ^*flox*^*Irx5*^*EGFP*^ (*Irx3/5* hypomorph) mutation: one *Irx3* allele harbors the null mutation, the other harbors loxP sequences, but is functional in the absence of Cre recombinase. *Irx5* includes EGFP sequences as in Fig 1A. (B) Fertility assessment of *Irx3/5* hypomorph mice through superovulation and *in vitro* fertilization (IVF). Number of oocytes retrieved after ovarian stimulation, number of 2-cell embryos 24 hours after fertilization with IVF and two-cell efficiency (number of 2-cell embryos divided by number of retrieved oocytes) are analyzed. Numbers are reported relative to the controls. (C) H&E histology of control and *Irx3/5* hypomorph ovaries. Red arrowhead: corpus luteum. Scale bar: 50 μm. (D) Quantification of ovarian structures for control (solid black bars) and *Irx3/5* hypomorph (hatched bars) ovaries. Control genotypes include 2 *Irx3*^*+*^*Irx5*^*+*^/*Irx3*^*+*^*Irx5*^*+*^ (Wild type), 3 *Irx3*^*Flox*^*Irx5*^*EGFP*^/*Irx3*^*+*^*Irx5*^*+*^ (Double Heterozygotes), and 1 *SF1Cre*^*Tg/+*^*;Irx3* ^*-*^*Irx5*^*EGFP*^/*Irx3*^*+*^*Irx5*^*+*^ (SF1Cre-Het) for a total of 6 control mice. Follicle numbers from each of these groups (derived from additional animals from another experiment not included in the IVF experiment and therefore, not included here) were not different from each other; therefore, they were combined into one control group. PD: primordial follicle; 1°: primary follicle; 2°: secondary follicle; PA: pre-antral follicle; A: antral follicle; TF: total follicles; AF-s: small atretic follicle; AF-l: large atretic follicle; CL: corpus luteum. Total follicle number includes follicles ranging from primordial to antral stage. Data in B and D represents the mean ± SEM. Statistics: two-sample t-test; **: p<0.01; *: p<0.05; #: p<0.2.

To facilitate evaluation of ovary development without embryonic lethality, we generated *Irx3*^*-*^*Irx5*^*EGFP*^/*Irx3*^*flox*^*Irx5*^*EGFP*^ mice (herein referred to as *Irx3/5* hypomorph) that maintain a single functional *Irx3* allele (flox; *Irx3*^*-/flox*^) on an *Irx5*-null background (*Irx5*^*EGFP/EGFP*^) (Fig 2A) [33]. Some of the *Irx3/5* hypomorph mice were viable, but they were too small and feeble to use in a standard breeding study (8-week-old body weights: *Irx3/5* hypomorph 15.14 +/- 1.11g; littermate controls 28.84 +/- 2.55g, p = 0.0003). Instead, we evaluated ovary function using superovulation and *in vitro* fertilization (IVF). By 8 weeks of age, the hypomorph mice were of sufficient size [34] to assess their fertility using this protocol. After superovulation, fragmented eggs were counted and then discarded and not included in IVF, but there was a higher fragmentation rate in *Irx3/5* hypomorph oocytes indicating poor quality of ovulated eggs (normalized fragmentation rate of 8.0 +/- 4.2 vs 1.0 +/- 0.1 for control oocytes, p = 0.15; S2 Fig). In addition, compared to controls, *Irx3/5* hypomorph females ovulated significantly fewer oocytes and therefore presented less 2-cell embryos (p < 0.05, Fig 2B, S2 Fig). The hypomorph mice also exhibited a trend of lower efficiency of progression to 2-cell embryos compared to the controls (p = 0.18, Fig 2B).

Fewer ovulation events in *Irx3/5* hypomorph mice was supported by the presence of fewer corpora lutea (CL) than control ovaries (Fig 2C). Quantification confirmed statistically fewer CLs in addition to reduced numbers of large atretic follicles in *Irx3/5* hypomorph compared to control ovaries (Fig 2D). No statistical difference was detected between groups for other follicle stages at this 8-week old time point (Fig 2D). *Irx3/5* hypomorph ovaries were smaller than controls (Fig 2C). This is explained by the substantial volume that CLs contribute to the size of control ovaries along with the disparity in body size. Altogether, these results suggest that growing follicles are unresponsive to ovulatory signals in *Irx3/5* hypomorph mice.

**Fig 3:**
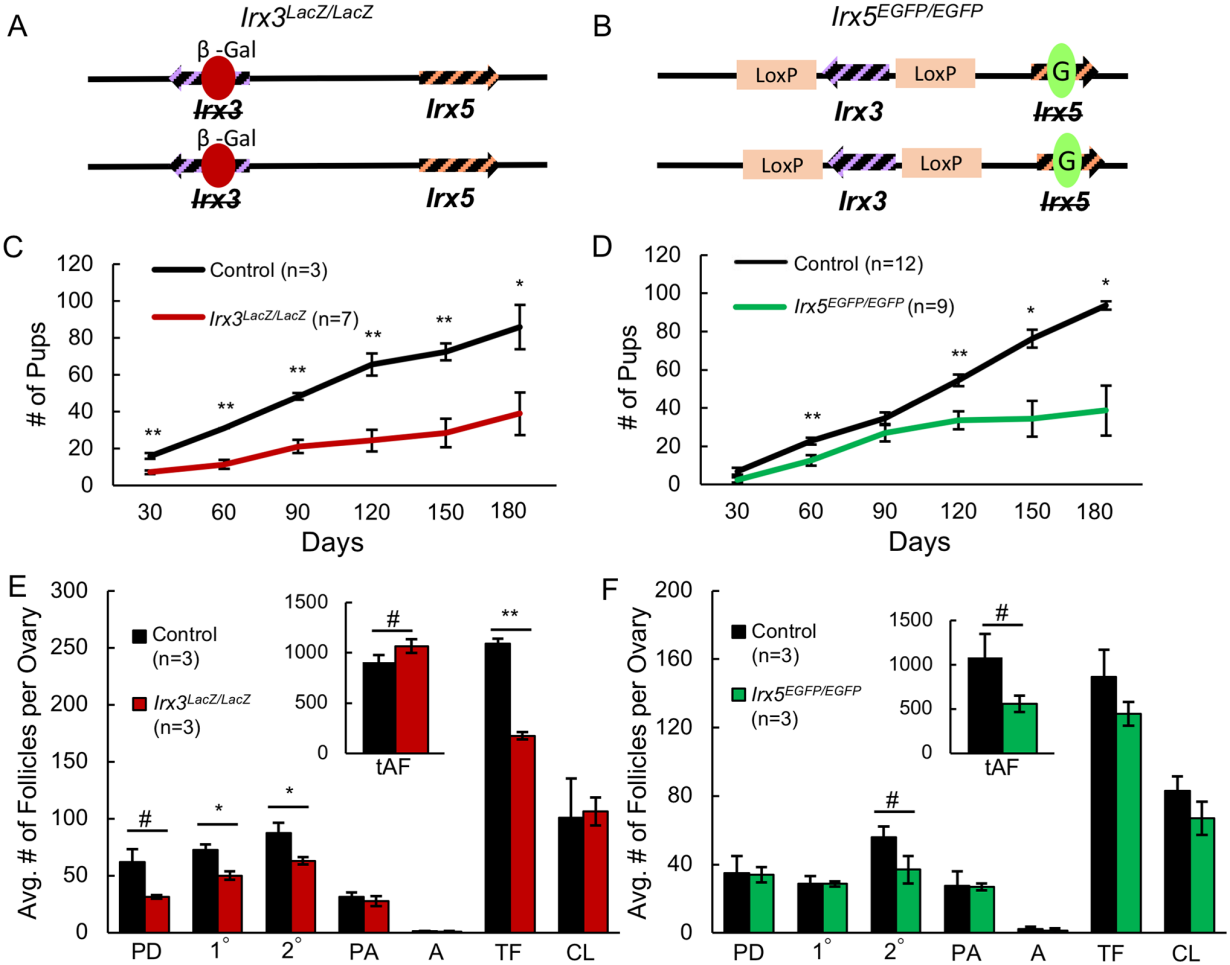
*Irx3*^*LacZ/LacZ*^ and *Irx5*^*EGFP/EGFP*^ mice are subfertile but with distinct breeding outcomes and ovarian morphology. (A) Schematic drawing of the *Irx3*^*LacZ/LacZ*^ mutation: *Irx3* null mutation is generated by the insertion of the reporter *LacZ* cassette expressing β-galactosidase (red oval with a β-Gal label) on both alleles while *Irx5* alleles remain intact. (B) Schematic drawing of the *Irx5*^*EGFP/EGFP*^ mutation: *Irx5* is rendered non-functional by the insertion of the EGFP sequence as in Figs 1A and 2A, while *Irx3* alleles contain loxP sequences but are functional in the absence of Cre recombinase. (C, D) Breeding study results representing the number of accumulated pups from *Irx3*^*LacZ/LacZ*^ females (C) and *Irx5*^*EGFP/EGFP*^ females (D) versus the controls during a 6-month (180-day) period. (E, F) Quantification of ovary structures from *Irx3*^*LacZ/LacZ*^ and *Irx5*^*EGFP/EGFP*^ and control ovaries. PD: primordial follicle; 1°: primary follicle; 2°: secondary follicle; PA: pre-antral follicle; A: antral follicle; tAF: total Atretic Follicles; CL: corpus luteum; tAF: total atretic follicle. Data in C-F represents the mean ± SEM. Statistics: two-sample t-test; *: p<0.05; **: p<0.01; ##: p<0.1; #: p<0.2.

Next, we examined the individual effects of *Irx3* and *Irx5* using *Irx3*^*LacZ/LacZ*^ and *Irx5*^*EGFP/EGFP*^ mice, respectively (Fig 3A and 3B). Each strain was previously documented to be robust and able to produce live offspring [33, 35, 36]. To assess their fertility, 6-week-old females from each strain were placed in a breeding study for six months. *Irx3*^*LacZ/LacZ*^ mice gave birth to fewer pups than their littermate controls throughout the breeding study (Fig 3C). *Irx5*^*EGFP/EGFP*^ mice also produced significantly fewer pups, but not until 120 days (about 4 months) into the breeding study (Fig 3D). Quantification of structures within ovaries after the conclusion of the breeding study supported that fertility defects were different between the two mutant strains. *Irx3*^*LacZ/LacZ*^ ovaries harbored significantly fewer total follicles caused by reduced contributions from young stage follicles and a trend of an increased number of atretic follicles, most of which were small atretic follicles (Fig 3E). Histological quantification of *Irx5*^*EGFP/EGFP*^ ovaries did not reveal any differences from the controls, except for a trend of relatively fewer secondary follicles and atretic follicles, both small and large (Fig 3F). Together, these results suggest that while *Irx3* and *Irx5* each contribute to fertility, their roles are potentially different. Further, when comparing the results between *Irx5*^*EGFP/EGFP*^, *Irx3*^*LacZ/LacZ*^ and the *Irx3/5* hypomorph (*Irx5*^*EGFP/EGFP*^ plus loss of one *Irx3* allele) strains, we conclude that defects associated with *Irx3* occur earlier and cause a more severe phenotype than the loss of *Irx5*.

### *Irx3* and *Irx5* exhibit distinct and dynamic expression profiles during ovary development

Because fertility studies suggested unique roles for *Irx3* and *Irx5*, we hypothesized that their expression profiles would be distinct. Previously, we reported that *Irx3* and *Irx5* initiate female-specific expression in developing gonads starting at E12.5. Both transcripts increase and then peak in ovaries around the time of birth (postnatal day 0, P0) followed by a rapid decline (S3 Fig) [23, 28, 29]. Because no reliable antibodies are currently available for IRX3 or IRX5, we used heterozygous *Irx3*^*LacZ/+*^ and *Irx5*^*EGFP/+*^ reporter mice [32, 37] to mark *Irx3* and *Irx5* expression, respectively, in specific cell types in the developing ovary over time. It is important to note that while each reporter reflects cellular localization over time, they do not reflect the endogenous subcellular localization of IRX3 or IRX5.

**Fig 4:**
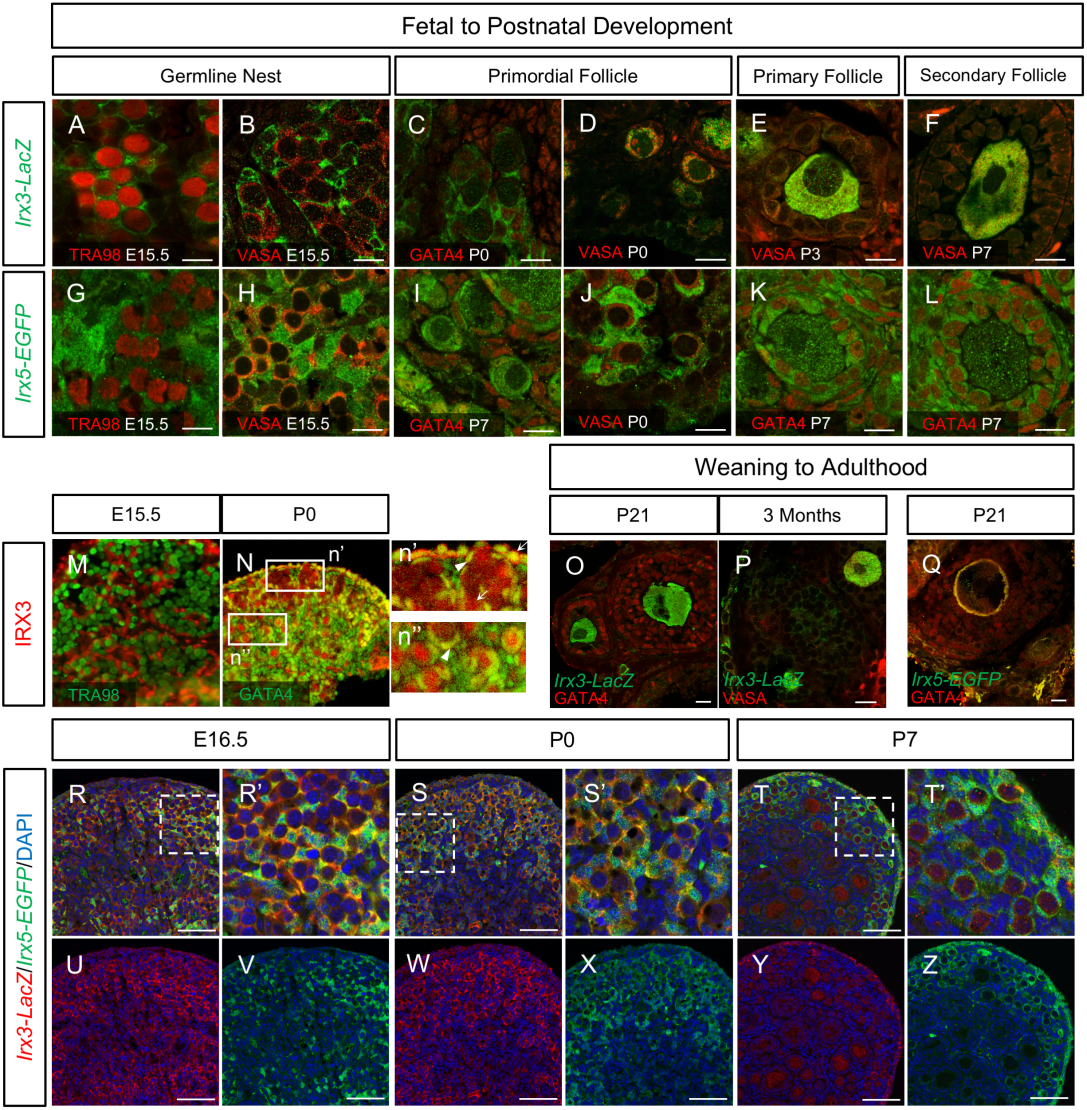
*Irx3* and *Irx5* exhibit dynamic cell type specific expression patterns during ovary development. (A-F) Immunofluorescence of *Irx3* (*Irx3-LacZ*, green) co-labeled with TRA98, a nuclear germ cell marker (red) or VASA, a cytoplasmic germ cell marker (red) and GATA4, a nuclear somatic cell marker (red) in follicles at various developmental stages. (G-L) Immunofluorescence of *Irx5* (*Irx5-EGFP*, green) with TRA98 (red), VASA (red) or GATA4 (red) in follicles across different developmental stages. (M, N) IRX3 (red) was examined using rabbit IRX3 antiserum antibody previously generated in our lab [28]. It is co-stained with TRA98 (green, M) and GATA4 (green, N). White boxed areas in N (n’ and n”) are enlarged to show germline nests and primordial follicles. White arrowheads indicate co-localization of GATA4 and nuclear IRX3. White arrows indicate cytoplasmic IRX3 expression. (O-Q) Post weaning and adult ovaries were examined for immunofluorescence of *Irx3-LacZ* (green, in O, P) and *Irx5-EGFP* (green, in Q) reporters with GATA4 (red) or VASA (red). (R-Z) Immunofluorescence co-staining of *Irx3-LacZ* and *Irx5-EGFP* in E16.5, P0 and P7 ovaries. White dashed boxes in R, S and T indicate areas of interest shown in R’, S’ and T’. Split channel images are presented in U-Z. Scale bars: 10 μm (A-L, O-Q); 50 μm (R-Z).

During fetal stages, cytoplasmic β-galactosidase staining, marking *Irx3* expression, was detected in GATA4-positive somatic cells but not TRA98- or VASA-positive germ cells of germline nests (S4 Fig, Fig 4A and 4B). While GATA4 marks all somatic cells, *Irx3*-*LacZ*-positive cells were associated only with somatic cells that surrounded germline nests (S4 Fig). As germline nests broke down and primordial follicles formed at birth, *Irx3*-*LacZ* expression was also detected in VASA-positive oocytes while being maintained in somatic cells (Fig 4C and 4D). Shortly after birth, strong *Irx3*-*LacZ* expression remained in oocytes of primary follicles, but its expression diminished and was eventually eliminated from granulosa cells (Fig 4E and 4F). Oocyte-enriched *Irx3-LacZ* expression was maintained throughout postnatal development and into adulthood (Fig 4O and 4P). Other members of the TALE homeodomain transcription factor family have been localized to both nuclear and cytoplasm [38, 39]. To evaluate subcellular localization of IRX3, we used antiserum against IRX3 that was previously made by our laboratory [28]. Results showed that during developmental stages, IRX3 was expressed within the nucleus of somatic cells that surrounded germline nests (Fig 4M). Just before germline nest breakdown, IRX3 expression was maintained within somatic cell nuclei, but was also detected in both cytoplasm and nuclei of germ cells within the germline nest and in the cytoplasm of ovarian surface epithelial cells (Fig 4N). Early-stage primordial follicles retained IRX3 expression in nuclei of pre-granulosa cells and in both nuclei and cytoplasm of germ cells. Finally, IRX3 was no longer detected in pre-granulosa cells, but was maintained in germ cells of more mature primordial follicles within the ovary medulla (Fig 4N). Unfortunately, this antiserum is no longer available. These data support the timeline of *Irx3* expression delineated by the reporter mouse strain and provide important new information. A summary of the subcellular localization of IRX3 over time in developing ovaries is presented in S1 Table.

*Irx5* is separated from *Irx3* by 550 kb on mouse chromosome 8 and shares a similar transcript expression profile with *Irx3* in several developing tissues [24, 25, 27], but cell specific localization varies depending on developmental stage and tissue type [32, 40]. Similar to *Irx3-LacZ*, *Irx5-EGFP* expression was absent in TRA98- and VASA-positive germ cells and was mostly restricted to GATA4-positive cells surrounding germline nests during fetal development (Fig 4G and 4H, S4 Fig). Further, during early postnatal days it was also detected (weakly) in oocytes of primordial and some primary follicles while being maintained in granulosa cells (Fig 4I and 4J). In contrast to *Irx3-LacZ*, oocyte expression of *Irx5-EGFP* diminished in later staged follicles, but its presence in GATA4-positive granulosa cells remained robust until at least P7 (Fig 4K and 4L). By P21, *Irx5-EGFP* was no longer detected in any cell type (Fig 4Q). There is no antibody available to evaluate subcellular localization of IRX5.

To validate *Irx3* and *Irx5* co-expression, we generated *Irx3*^*LacZ*^*Irx5*^*+*^/*Irx3*^*flox*^*Irx5*^*EGFP*^ dual-reporter mice. In support of the previous results, double immunofluorescence with β-galactosidase and EGFP antibodies indicated that both *Irx3* and *Irx5* were expressed in somatic cells outlining germline nests at E16.5 (Fig 4R, 4R’, 4U and 4V). Upon germline nest breakdown and primordial follicle formation at birth (P0), *Irx3* and *Irx5* were co-expressed in pre-granulosa cells at the ovarian cortex with decreased signal intensity for both in the medulla (Fig 4S, 4S’, 4W and 4X). By P7, their expression patterns were distinct with *Irx3* and *Irx5* confined to oocytes and granulosa cells, respectively (Fig 4T, 4T’, 4Y and 4Z). In addition, both *Irx3* and *Irx5* were detected in the ovarian surface epithelial cell layer at all three time points (Fig 4R–4Z).

**Fig 5:**
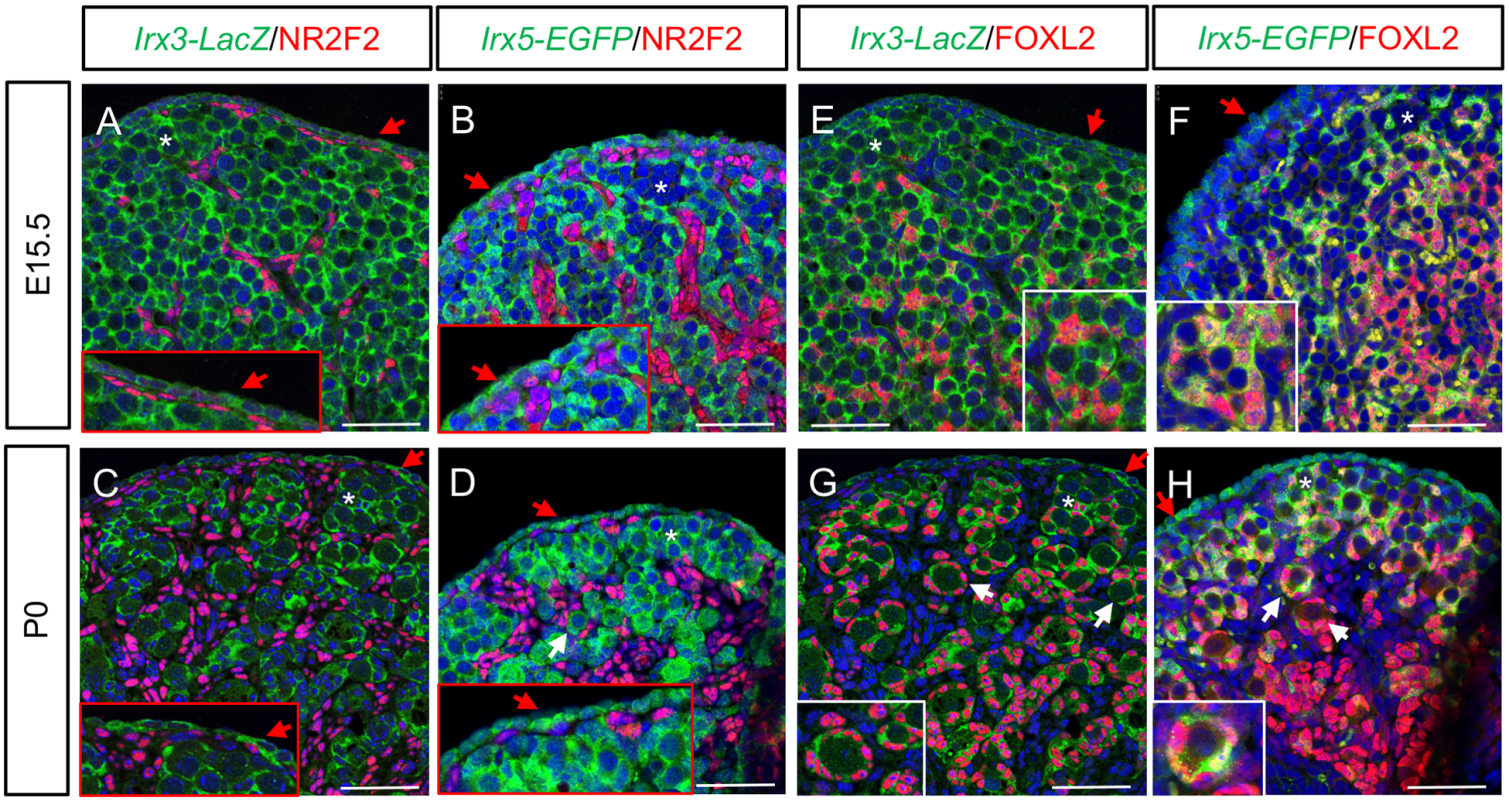
*Irx3* and *Irx5* mark the pre-granulosa cell subpopulation of somatic cells. (A-D) Double immunofluorescence of NR2F2 (red) with *Irx3-LacZ* (A, C) or *Irx5-EGFP* (B, D) in E15.5 and P0 ovaries. (E-H) Double immunofluorescence of FOXL2 (red) with *Irx3-LacZ* (E, G) or *Irx5-EGFP* (F, H) in E15.5 and P0 ovaries. Blue: DAPI stain. Asterisk (*): germline nest; white arrow: primordial follicle; red arrow: ovarian surface epithelium. Scale bars: 50 μm.

At least three different populations of somatic cells have been reported in the developing ovary [41]. To identify the specific somatic cell populations for *Irx3* and *Irx5* expression, we evaluated whether their reporters co-localized with known ovarian somatic cell markers including the vasculature-associated somatic cell marker, COUP-TFII (NR2F2), and the pre-granulosa cell marker, FOXL2 [14, 41]. Results from ovaries at E15.5 and P0 showed that both *Irx3* and *Irx5* were not expressed in NR2F2-positive cells (Fig 5A–5D). Co-expression with FOXL2-positive cells was more nuanced. FOXL2 was detected in *Irx3-* and *Irx5*-positive somatic cells in the medulla at E15.5 but not in the cortex (Fig 5E and 5F). By P0, all pre-granulosa cells expressed FOXL2 (Fig 5G and 5H). While *Irx3* was detected in all FOXL2-positive cells though weaker in the medulla (Fig 5G), *Irx5* expression co-localized with FOXL2-positive cells only within the cortex and was absent in the medulla (Fig 5H). As described above (Fig 4R–4Z), *Irx3* and *Irx5* were expressed in the ovarian surface epithelium, which was negative for FOXL2 and NR2F2 at both E15.5 and P0 (Fig 5A–5H).

### Defective granulosa cell function and impaired cell-cell interactions cause *Irx3/5* DKO follicle death

Thus far, we have demonstrated that *Irx3* and *Irx5* both contribute to female fertility and that ovarian function is increasingly impaired as alleles of *Irx3* are eliminated in the context of the *Irx5*-null mouse. The most severe phenotype is presented in *Irx3/5* DKO ovarian grafts with follicles at varying stages of cell dysfunction and death (Fig 1) [3]. After having determined that *Irx3* and *Irx5* are expressed in both cell types that make up a follicle, we next set out to identify potential mechanisms to explain defects in mutant ovaries.

**Fig 6:**
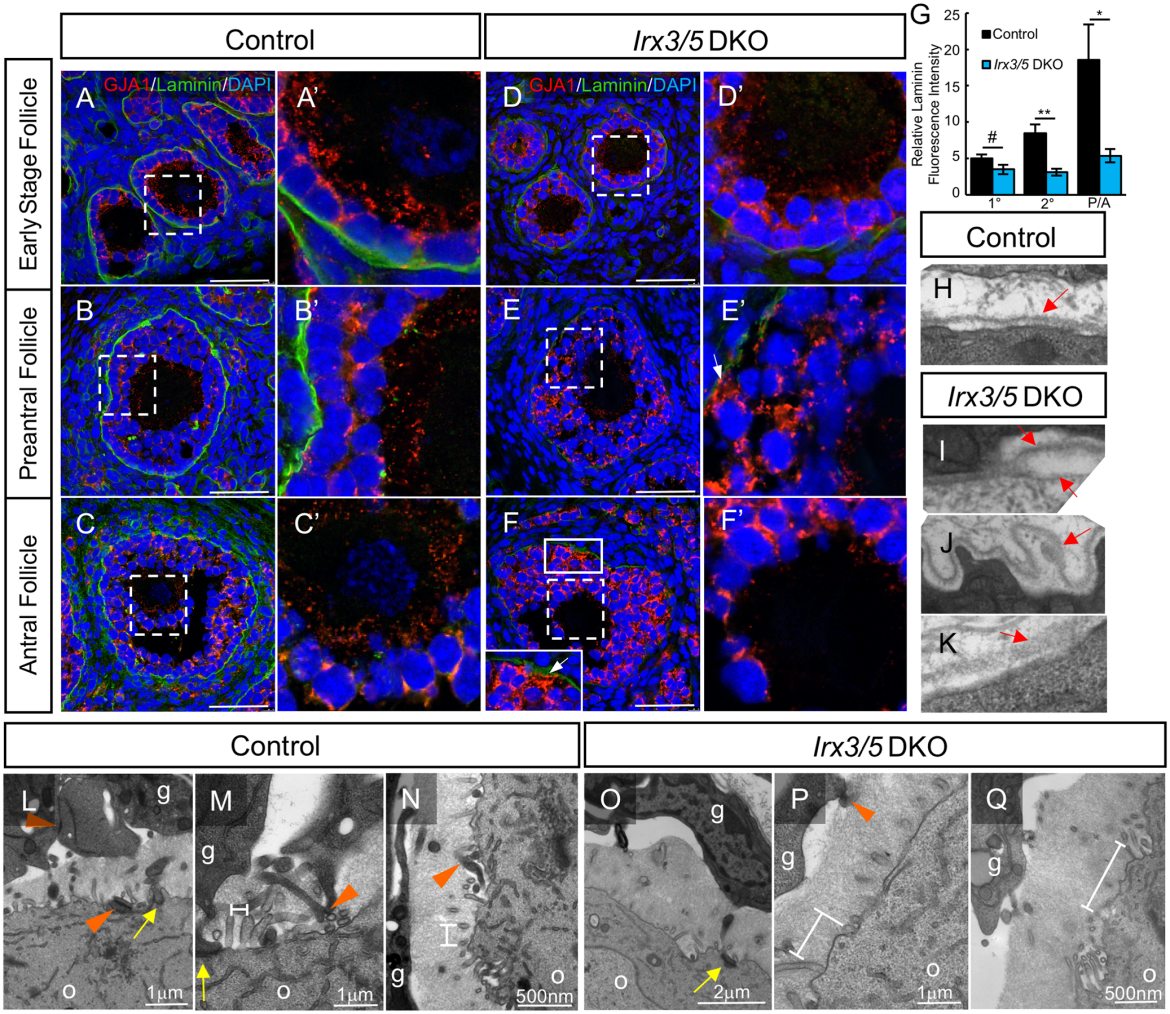
*Irx3/5* DKO follicles exhibit abnormal basement membrane deposition, GJA1 misexpression and reduced granulosa cell-oocyte interactions. Ovarian follicles of 2-week (A, A’, D, D’ and G-P) and 3-week (B-C’ and E-F’) KCT grafts were examined for follicle integrity. (A-F’) Immunofluorescence of gap junction protein 1 (connexin 43, GJA1, red) and basement membrane marker, laminin (green) in control and *Irx3/5* DKO follicles. White dashed boxes in A-F indicate area of interests shown in A’-F’. The solid white box in F is enlarged in the inset. White arrow: GJA1 expression facing the basement membrane. (G) Quantification of relative laminin fluorescence intensity of primary to antral stage follicles in 2-week and 3-week KCT grafts. Data represents Mean ± SEM. Statistics: two-sample t-test; *: p<0.05; **: p<0.01; #: p<0.2. (H-K) Transmission electron micrographs of the basement membrane from control (H) and *Irx3/5* DKO (I-K) primary follicles. Red arrows: basement membrane. (L-Q) Transmission electron micrograph of the zona pellucida interaction zone between the oocyte and granulosa cells of control (L-N) and *Irx3/5* DKO (O-Q) ovaries. g: granulosa cell; o: oocyte; orange arrowhead: granulosa cell-granulosa cell contact; yellow arrow: granulosa cell-oocyte contact; double-sided bar: distance between two adjacent oocyte microvilli. Scale bars: 50 μm (A-F); 1 μm (L, M, P); 2 μm (O); 500 nm (N, Q).

Potential causes for granulosa cell dysfunction include loss of cell identity or defective cell polarity/orientation within the follicle structure. Immunofluorescence results showed that *Irx3/5* DKO granulosa cells maintained their identity as expression of the granulosa cell markers, anti-Müllerian Hormone (AMH) and FOXL2, was not different from controls, and no upregulation of SOX9 expression was detected by 2 weeks post KCT surgery (S5 Fig). Instead, immunofluorescence and TEM approaches suggested a loss of granulosa cell polarity or orientation (Fig 6). Granulosa cells produce and guide deposition of the basement membrane that surrounds each follicle, which in turn influences their proliferation and follicle growth [42–44]. Evaluation of laminin, a basement membrane marker, demonstrated that a continuous basement membrane surrounded follicles in mutant and control tissues; however, the intensity of its expression was reduced in *Irx3/5* DKO follicles, especially as they matured (compare Fig 6A–6C and 6D–6F, quantified in Fig 6G). Laminin expression was detected in primary and early secondary follicles but diminished in later staged follicles (compare Fig 6D, 6E and 6F). This finding was supported by results that showed no difference in laminin transcript levels at early developmental stages of E13.5 or 7d KCT (S6 Fig). Closer examination by TEM showed that in contrast to the smooth, organized basement membrane adjacent to wild type granulosa cells (Fig 6H), the structure in *Irx3/5* DKO tissues presented with a variety of phenotypes. We observed increased incidences of abnormal membrane deposition including double layers (Fig 6I), abnormal production with looping (Fig 6J), and diffuse, permeable deposition (example, Fig 6K). The incidences of these abnormalities are outlined in S2 Table.

Granulosa cells must communicate with each other, primarily via connexin 43 (Gap junction protein 1, GJA1), to integrate signals that promote their proliferation, differentiation, and survival [45, 46]. In addition, *Irx3* is known to repress *Gja1* transcription in neonatal ventricular myocytes [37]. Although there was no difference in transcript accumulation for *Gja1* between wild type and mutant ovaries at earlier stages including E13.5 or 7d KCT (S6 Fig), our immunofluorescence analysis uncovered abnormal protein localization patterns that varied depending on follicle stage. In early stage follicles (transitioning primordial and early primary follicles), GJA1 was detected as expected between granulosa cells, but also at the granulosa cell—oocyte interface in mutant tissues (S7 Fig). Later, starting at the late primary follicle stage, oocytes synthesize the zona pellucida, a specialized extracellular matrix that plays critical roles in oocyte growth, fertilization, and early embryonic development. Simultaneously, granulosa cells develop transzonal processes that are designed to traverse the zona pellucida to extensively interact with each other and the oocyte [45, 47]. Robust GJA1 expression was detected within the zona pellucida of late primary and more mature follicles in control tissues (Fig 6A–6C and 6A’–6C’). In contrast, GJA1 signal decreased in intensity in this region as follicles matured in *Irx3/5* DKO ovaries indicating gradually diminishing interactions between transzonal processes over time (Fig 6D–6F and 6D’–6F’). In support of GJA1 data, TEM images validated the decrease in transzonal projections from different granulosa cells. While frequent interactions were observed among the trans-zona in control follicles (Fig 6L–6N), few were observed in *Irx3/5* DKO follicles (Fig 6O–6Q). In other areas of *Irx3/5* DKO antral follicles, populations of granulosa cells exhibited abnormal accumulation of GJA1, including in cell membranes at the interface with the basement membrane (compare Fig 6B’ to 6E’ and 6F).

Finally, TEM uncovered a generalized paucity of interactions in the zona pellucida between the oocyte and granulosa cells (Fig 6L–6Q). Besides distinctly fewer transzonal processes extending from granulosa cells, the organization of structures originating from the oocyte was also different in *Irx3/5* DKO follicles. Widened and inconsistent distances separated microvilli extensions from *Irx3/5* DKO oocytes compared to controls and there were fewer microvilli in many cases (Fig 6M, 6N, 6P and 6Q). Taken together, both cell types contribute fewer interacting processes that result in fewer opportunities for cell-cell communication within the interacting zone of *Irx3/5* DKO follicles. To test whether the disrupted communication was the cause or the result of follicle death, we tested for apoptosis using immunofluorescence with antibodies against cleaved caspase 3 and laminin on wild type (n = 6) and *Irx3/5 DKO* (n = 5) grafts evaluating a range of 2–16 follicles per slide. Cleaved caspase 3-positive cells were rare in both wild type and *Irx3/5 DKO* follicles indicating that these disruptions occur before follicle death (S8 Fig). Taken together, we conclude that *Irx3* and *Irx5* function in oocytes and granulosa cells to promote directional intercellular interactions along with a functional follicle-ECM niche to ensure follicle survival.

## Discussion

It has long been established that somatic cell-oocyte communication is critical for follicle integrity and promotes healthy ovarian development and function. During ovarian development, factors are expressed in specific spatio-temporal fashion to not only determine oocyte and somatic cell identity, but also coordinate cell-cell interactions that ensure proper breakdown of germline nests and establishment of the primordial follicle pool for future recruitment [7–13, 17, 48]. The mechanisms by which cell-cell communication networks are established between pre-granulosa cells and oocytes within new primordial follicles, however, remain unknown. We previously showed that *Irx3* and *Irx5* transcripts peaked in the ovary around birth and that their expression was required to maintain oocyte-granulosa cell contacts within early-staged follicles [3, 23]. The objective for this study was to investigate roles for *Irx3* and *Irx5* in ovarian development and function. We demonstrated that female fertility was significantly impaired in both *Irx3*^*LacZ/LacZ*^ and *Irx5*^*EGFP/EGFP*^ single mutant mice. Their subfertility phenotypes were different, suggesting their distinct roles. In support, careful analysis of *Irx3* and *Irx5* expression profiles highlighted primordial follicle formation as a critical time point when their expression patterns transitioned from shared to unique cell localization. Evaluation of *Irx3/5* DKO follicles underlined the importance of their individual expression patterns. Most follicles exhibited abnormal morphology within early postnatal stage *Irx3/5* DKO ovarian tissue. Evidence points to defective granulosa cell function and granulosa cell-oocyte communication structures as a cause of follicle death. Based on these data, we conclude that *Irx3* and *Irx5* direct construction of the communication infrastructure within and between pre-granulosa cells and the oocyte as nascent primordial follicles form.

### Dynamic expression of *Irx3* and *Irx5* precedes and is maintained as intercellular communication networks are established within nascent follicles

It has been proposed that two distinct populations of primordial follicles exist in the postnatal ovary that are activated in two separate waves to play different roles in ovarian development and fertility [49, 50]. *Foxl2* and *Lgr5* are reported to mark pre-granulosa cell populations that distinguish the two pools of primordial follicles [41, 48, 51]. We propose that *Irx3* and *Irx5* are present in both populations. During embryonic development (E15.5), *Irx3* and *Irx5* are expressed in the ovarian surface epithelium and in somatic cells that outline germline nests, whereas FOXL2 expression is restricted to nests located in the ovarian medulla. These findings support previous reports that FOXL2 marked early somatic cells within the medulla that give rise to granulosa cells within primordial follicles that are activated during the first wave, immediately after birth [48, 50]. Mork and colleagues suggested a second wave of primordial follicles that established the lifetime ovarian reserve. These follicles incorporated pre-granulosa cells that originated from FOXL2-negative ovarian surface epithelial cells, which would eventually mature into FOXL2-positive granulosa cells [48]. These ovarian surface epithelial cells were found to be *Lgr5*-positive, and they matured into pre-granulosa cells of primordial follicles that populated the cortex to define the ovarian reserve at birth [41, 52, 53]. Our results show that *Irx3* and *Irx5* are expressed in ovarian surface epithelium (*Lgr5*-positive) and both FOXL2-negative (presumably *Lgr5*-positive) and FOXL2-positive pre-granulosa cells, suggesting that *Irx3* and *Irx5* mark cells destined to be granulosa cells within follicles assembled for both populations of primordial follicles.

**Fig 7:**
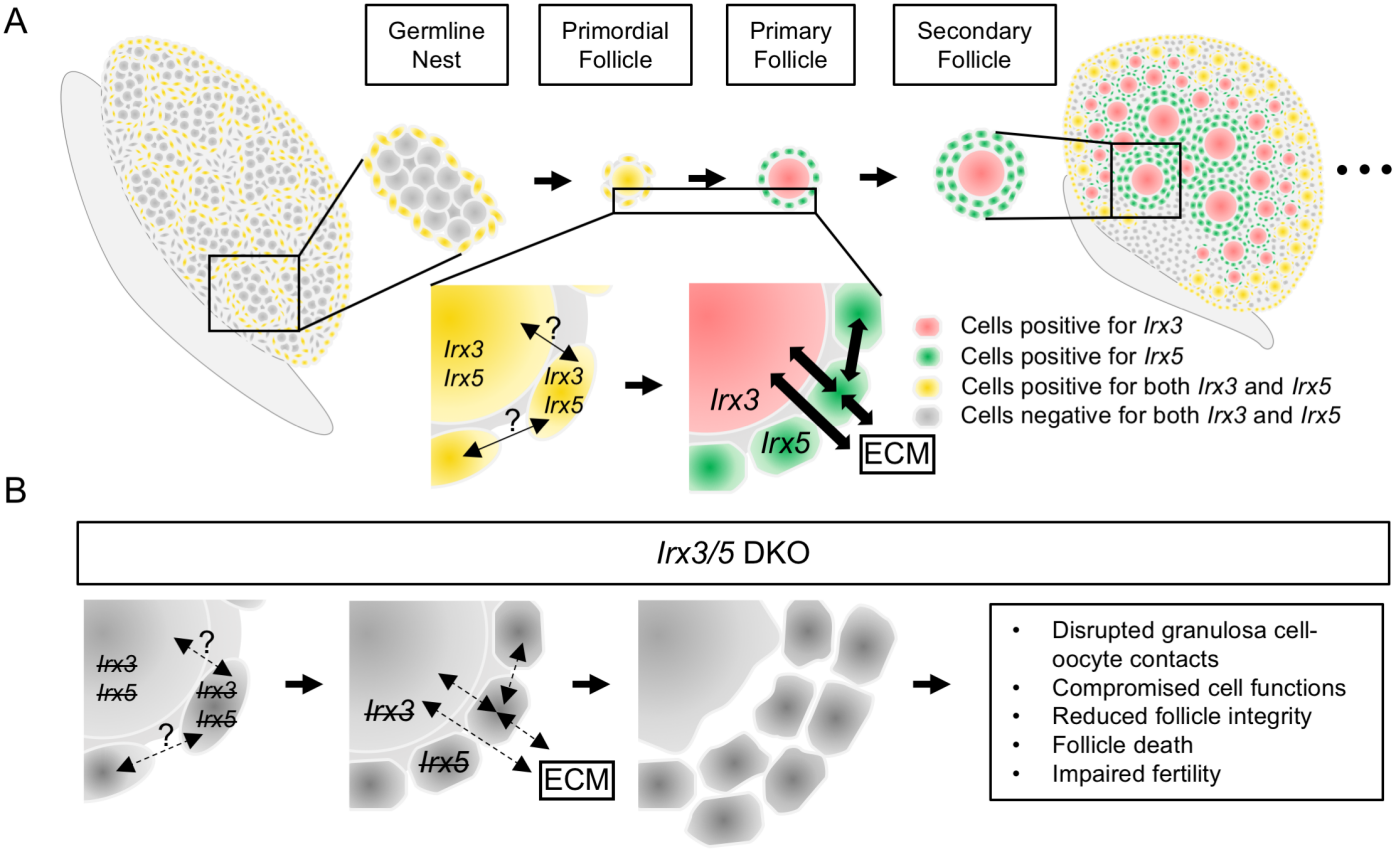
A model for *Irx3* and *Irx5* functions within the ovary. (A) *Irx3* and *Irx5* exhibit dynamic expression patterns in the ovary throughout development. During the primordial follicle formation stage, *Irx3* and *Irx5* work together in both granulosa cells and the oocyte to promote establishment of cell-cell communication networks. When primordial follicle begins to mature, *Irx3* and *Irx5* collaborate between oocyte and granulosa cells respectively to promote the interactions between granulosa cells, granulosa cells and oocyte, granulosa cells and extracellular matrix (ECM) components, and the oocyte and ECM components. (B) Null expression of *Irx3* and *Irx5* in *Irx3/5* DKO follicles compromises follicle formation, which leads to weakened interactions among oocyte, granulosa cells and ECM to result in disrupted follicle integrity and reduced fertility.

Surprisingly, *Irx3* and *Irx5* were found to be expressed briefly in oocytes while they were still in pre-granulosa cells during the perinatal stage when germline nests break down and primordial follicles form. As follicles develop beyond the primordial and primary stages, *Irx3* expression was detected only in oocytes while *Irx5* expression was restricted to granulosa cells (summarized in the model in Fig 7A). Notably, evaluation with IRX3 antiserum indicated that IRX3 was expressed in the nucleus and cytoplasm of the oocyte whereas pre-granulosa cell expression was confined to the cell nucleus (S1 Table). The divergence of *Irx3* and *Irx5* expression patterns occurs as follicles transition from primordial to primary follicles, which represents an event of follicle development of which little is known. The shared function of Iroquois family factors, including IRX3 and IRX5, is to promote patterning during development [25, 27, 33, 54]. Other members of the TALE homeodomain transcription factor family are known to shuttle between the nucleus and cytoplasm in certain cells at specific time points during development. Notably, when these factors were detected in the cytoplasm, they co-localized with cytoskeletal proteins (actin, non-muscle myosin) [38, 39]. The activity of these factors in the cytoplasm is still under investigation, but there have been links to their facilitating responses of extracellular signals. That *Irx3* and *Irx5* are expressed in two distinct but neighboring cell types suggest their cooperative role in orchestrating cell-cell interactions. Indeed, studies in heart development revealed that *Irx3* and *Irx5* performed both supportive and antagonistic functions during heart development and in postnatal cardiac electrophysiology induction, respectively [32]. When *Irx3* and *Irx5* were not co-expressed, they worked together via different but spatially connected cells to promote electric propagation [32]. In support of studies within other developing organs, we find that *Irx3* and *Irx5* impart spatial expression patterns that transitions at the critical stage of primordial follicle formation.

### *Irx3* and *Irx5* synchronize oocyte and granulosa cell interactions within new primordial follicles to promote lifelong follicle health

*Irx3*^*LacZ/LacZ*^ and *Irx5*^*EGFP/EGFP*^ mutant mice have been used to investigate functions of other systems and are healthy and fertile [33, 35, 37, 55]. Six-month breeding studies, however, revealed that both strains accumulated ≤ 50% pups compared to their littermate controls, but in different patterns. *Irx3*^*LacZ/LacZ*^ females consistently produced fewer pups throughout the entire study. At 8 months of age, *Irx3*^*LacZ/LacZ*^ ovaries harbored fewer early stage follicles along with a trend for increased atretic follicles compared to controls suggesting that fewer oocytes were available for recruitment. At this rate, it is likely that *Irx3*^*LacZ/LacZ*^ mutants would deplete their ovarian reserve much earlier than the controls, as in POI cases. Furthermore, we demonstrated that *Irx3* was present in the oocyte beyond the primordial follicle stage. These data, along with increased oocyte fragmentation detected in *Irx3/5* hypomorph mice suggest that *Irx3* impacts oocyte quality and therefore, fertilization success. These outcomes, together with its transitioning expression profile, suggest that *Irx3* is important in both oocytes and granulosa cells.

*Irx5*^*EGFP/EGFP*^ mutant females accumulated the same number of offspring as controls for the first three months but failed to increase their numbers thereafter. Thus, it was somewhat surprising to find no obvious difference in follicle numbers between groups. Likewise, *Irx3/5* hypomorph females that lack one *Irx3* and both *Irx5* alleles ovulated significantly fewer eggs, but ovary histology did not differ from the controls other than an expected reduction in the number of CLs. Successful ovulation requires response and coordination of granulosa cells [22]. These fertility defects, along with the knowledge that all *Irx5* expression is eliminated before puberty suggest that during the perinatal stage, *Irx5* prepares granulosa cells for future responsiveness to external signals required for maturation and ovulation. This process is substantially more successful when *Irx5*-positive granulosa cells can communicate with oocytes harboring their full complement of *Irx3* expression. It is important to note however, that there remains a possibility that decreased fecundity may also be caused by implantation defects. This is currently under investigation. Altogether, these results are similar to that of *Bmp15*^*-/-*^ ovaries that exhibited impaired ovulatory function with generally normal morphology except for evidence of trapped oocytes [56, 57]. Both mutant mouse models support the need for the early onset of communication between cell types in the growing follicle.

Complete loss of both *Irx3* and *Irx5* has devastating effects on follicle health much earlier than the other *Irx* mutant mouse models. We found that *Irx3/5* DKO follicles deposited basement membrane sections with abnormal morphology and that it appeared to diminish as follicles matured. Previous *in vitro* studies have demonstrated that extracellular matrix (ECM) proteins are critical for follicle survival [58]. As such, ultrastructure images provided evidence that some follicles, though generally healthy looking, contained less competent oocytes when they were surrounded by looped or multiple layers of basement membrane deposits and the oocytes progressed through atresia when basement membrane became fragmented [59]. Laminin is a major ECM component of the basement membrane surrounding follicles that is produced by granulosa cells, among other cells, suggesting that they contribute towards follicle basement membrane organization [60–62]. Thus, the presence of insufficient follicular basement membranes in *Irx3/5* DKO follicles suggest that *Irx3* and *Irx5* are required for granulosa cell function. Moreover, it has been proposed that oocytes regulate basement membrane deposition via oocyte-specific glycoprotein signals through theca cells [63]. Therefore, we should include the possibility that the oocyte contributes to defects observed in *Irx3/5* DKO follicles, especially given the presence of *Irx3* in oocytes.

More alarming than basement membrane insufficiency was the disarray of cell-cell communication at multiple levels. *Irx3/5* DKO follicles exhibited mis-localized and abnormal accumulation of GJA1 providing further support that granulosa cells failed to establish functional communication ports. Polarized junction protein expression is an indication of normal granulosa cell function and is required to establish appropriate cell-cell communication [45, 64]. In addition, in *Irx3/5* DKO follicles, GJA1 expression was nearly absent in the region of the zona pellucida of pre-ovulatory-stage follicles suggesting few granulosa cell-granulosa cell interactions near the oocyte. This was supported by TEM images that validated the lack of granulosa cell-derived transzonal processes, but also highlighted abnormal patterns of microvilli extensions from the oocyte. Together, these data highlight roles in both granulosa cell and oocyte for *Irx3* and *Irx5* in maintaining follicle integrity.

Based on the data reported here, we propose a model to illustrate our conclusion that *Irx3* and *Irx5* work together, in the same and then in neighboring cells (pre-granulosa cells and oocyte) over time to determine the pattern, polarity, and therefore, the foundation by which communication networks are established within new follicles. This model is remarkably similar to that proposed by Gaborit et al. in which *Irx3* and *Irx5* have complex interactions in the developing heart. During heart development, they exhibit redundant activity as they colocalize in the endocardium. Thereafter, in the postnatal heart, their expressions transition to neighboring cells where they modulate the expression of intercellular channels to maintain appropriate conductance [32]. Fig 7A models the expression profiles for each factor over time with the transition into cell-specific expression patterns that coincides with the establishment of the ovarian reserve in the form of primordial follicles. We propose that cytoplasmic IRX3 or IRX5 interacts with cell cytoskeleton to direct polarity and organize cell extensions. Further, these cells are simultaneously producing and organizing ECM components to establish the niche for the new follicle. These parameters are established in the perinatal ovary, but their effects continue to impact follicular health throughout the reproductive lifespan of the ovary. Fig 7B highlights the impact of the *Irx3/5* DKO mutation. Although follicles have formed with a single oocyte surrounded by granulosa cells, the cell-cell and cell-ECM interactions have not been established for lasting quality. Instead, cell-cell interactions become compromised and eventually, the oocyte and granulosa cells lose contact and die.

The biggest challenge for our study was the embryonic lethality of *Irx3* and *Irx5* double mutation. With the generation of *Irx3/5* hypomorph and *Irx3* and *Irx5* single mutant females, we gained insight into factor-specific roles. The reporter lines were critical to highlight the potential for time- and cell-specific effects. Thus, the results of this current study provided a map for future investigations of *Irx3* and *Irx5* functions in the ovary using cell specific mutations. Furthermore, the mis-expression of GJA1 and abnormal basement membrane in *Irx3/5* DKO follicles render it necessary to investigate the interactions of *Irx3* and *Irx5* with their downstream targets and other components of cellular architecture. Here, we show convincing evidence that *Irx3* and *Irx5* are critical factors for the developing ovary while they are not expressed in the developing testis. In the ovary, they work together to mark future granulosa cells during the fetal stage, and then function within both the oocyte and its protective somatic cell layer to coordinate formation of the nascent primordial follicles. Their legacy is manifest in functional oocyte-granulosa cell interactions that ensure healthy follicle maturation and competent oocytes.

## Materials and methods

### Ethics statement

Adult animals were euthanized by CO2 asphyxiation followed by cervical dislocation. Embryonic pups were euthanized by decapitation with a razor blade. Animal housing and all procedures described were reviewed and approved by the Institutional Animal Care and Use Committee at the University of Wisconsin—Madison and were performed in accordance with National Institute of Health Guiding Principles for the Care and Use of Laboratory Animals.

### Animals

Mouse strains included CD1 outbred mice (Crl:CD1(ICR), Charles River, MA), nude mice (Crl:NU-*Foxn1*^*nu*^, Charles River, MA), *Irx3*^*LacZ*^ [37], *Irx3*^*flox*^*Irx5*^*EGFP*^ (referenced as *Irx5*^*EGFP*^ here) [32], and *Irx3*^*-*^*Irx5*^*EGFP*^ [32], all of which were maintained on a CD1 genetic background. Genotyping for *Irx3*^*LacZ*^, *Irx5*^*EGFP*^ and *Irx3*^*-*^*Irx5*^*EGFP*^ was carried out as previously reported [32, 37]. Timed mating was identified by the presence of a vaginal plug, which was designated as embryonic day 0.5 (E0.5). Ovaries were collected at the indicated time points for further analysis.

### Kidney capsule transplant (KCT) surgery

Gonads were harvested from embryonic day (E) E12.5 embryos resulting from the breeding of *Irx3*^*-*^*Irx5*^*EGFP*^/*Irx3*^*+*^*Irx5*^*+*^ male and female mice. Post-genotyping, *Irx3*^*+*^*Irx5*^*+*^/*Irx3*^*+*^*Irx5*^*+*^ (wild-type, WT) and *Irx3*^*-*^*Irx5*^*EGFP*^/*Irx3*^*-*^*Irx5*^*EGFP*^ (*Irx3/5* DKO) ovaries were transplanted under the kidney capsule of an ovariectomized nude mouse of at least eight-week-old as previously described [23]. The nude mice were ovariectomized at least 2 weeks prior to KCT surgeries. Grafted ovaries were recovered 10 days, 2 weeks or 3 weeks post transplantation.

### Superovulation

*Irx3*^*-*^*Irx5*^*EGFP*^/*Irx3*^*Flox*^*Irx5*^*EGFP*^ (*Irx3/5* hypomorph) pups often died shortly after birth. Those that survived were the same size as littermate controls at first, but growth was significantly retarded; therefore, they were maintained with their dam to encourage survival with continued access to milk. Once pups reached sufficient size to respond to hormone induction (approximately 8 weeks of age)[34], they and their littermate (age matched) controls were subjected to a superovulation protocol including intraperitoneal (IP) injections with 5 IU each of pregnant mare’s serum gonadotropin (PMSG) and human chorionic gonadotropin (hCG) 64 hours and 16 hours, respectively, prior to oocyte harvest. Oocytes were extracted at the Transgenic Animal Facility at UW-Madison Biotechnology Center and *in vitro* fertilized with wild type sperm. The number of ovulated oocytes, fragmented oocytes, oocytes used for IVF and 2-cell embryos post fertilization were recorded. Due to variability across superovulation and IVF experiments, each of the numbers were normalized relative to the control data within the same experiment.

### Breeding study

*Irx3*^*LacZ/LacZ*^, *Irx5*^*EGFP/EGFP*^ females and their respective littermate control females were set up with wild type males to breed for 6 months. WT males were replaced and rotated at least once. The litter sizes and birth dates were closely monitored and recorded throughout the breeding period.

### Histology

Ovaries were harvested from each experimental paradigm, fixed in 4% PFA overnight and then embedded in paraffin. Paraffin blocks were sectioned at 8 μm thickness and then stained with hematoxylin and eosin (H&E) for histological analysis.

### Follicle quantification

Ovaries were sectioned completely through and every tenth section was used to quantify the numbers for each structure. Corpora lutea (CLs) and large follicles were counted in each quantified section for each ovary; therefore, these structures were counted multiple times resulting in the high numbers/ovary. All counting was completed by investigators blinded to ovary genotype.

### Immunofluorescence

Mouse ovaries were harvested at E15.5 and P0, P3, P7, P21 and 8 months, fixed in 4% paraformaldehyde (PFA) in phosphate buffer saline (PBS) at 4°C overnight, and then washed in PBS. Samples were dehydrated through an ethanol gradient, cleared in xylene and then embedded in paraffin. Ovary grafts used for GJA1 and laminin staining were snap-frozen in Tissue-Plus O.C.T. Compound embedding medium immediately after dissection and were fixed in acetone for 10 minutes after sectioning prior to storage. Primary antibodies were applied to 8 μm paraffin tissue sections and 8 μm frozen sections and then incubated at 4°C overnight (Table 1). Secondary antibodies (Table 2) were then incubated at room temperature for 1 hour. A 10X DAPI (4’,6-diamidino-2-phenylindole) in PBS solution (1:500) was used as a nuclear counterstain. Images were collected on a Leica SP8 confocal microscope and a Keyence BZ-X700 microscope at the School of Veterinary Medicine, University of Wisconsin—Madison. Other images were acquired using a Nikon C1 confocal microscope at the Monash Micro Imaging Facility and on a Zeiss LSM800 confocal microscope at the Biological Optical Microscopy Platform (BOMP) at the Department of Anatomy and Neuroscience, the University of Melbourne. Images were processed with ImageJ or Adobe Photoshop.

**Table 1:** Primary antibodies.

Primary Antibody Name	Source	Catalog #	Dilution
Goat anti GATA4	Santa Cruz Biotechnology	sc-1237	1:100
Biotin anti GFP	Abcam	ab6658	1:50
Chicken anti GFP	Abcam	ab13970	1:100
Mouse anti GFP	Santa Cruz Biotechnology	sc-9996	1:100
Rabbit anti DDX4 (VASA)	Santa Cruz Biotechnology	sc-67185	1:50
Chicken anti β-galactosidase	Abcam	ab9361	1:100
Rabbit anti FOXL2	[65]		1:400
Mouse anti NR2F2	Perseus Proteomics	PP-H7147-00	1:300
Rabbit anti GJA1	Abcam	ab11370	1:250
Goat anti Laminin	Abcam	ab11575	1:250
Goat anti FOXL2	Abcam	ab5096	1:150
Rabbit anti MIS	Santa Cruz Biotechnology	sc-6886	1:250
Goat anti SOX9	Abcam	ab26414	1:500
Rabbit anti Cleaved Caspase 3	Cell Signaling Technology	9661	1:250
Rabbit anti-IRX3 serum	[28]	N/A	1:250

**Table 2:** Secondary antibodies.

Secondary Antibody Name	Source	Catalog #	Dilution
Alexa Fluor 488-conjugate IgG fraction monoclonal mouse anti-biotin	Jackson ImmunoResearch	200-542-211	1:100
Immuno-pure goat anti-rabbit IgG Rhodamin conjugated	Pierce	31670	1:100
Immuno-pure rabbit anti-goat IgG Rhodamin conjugated	Pierce	31650	1:150
Alexa Fluor 546 donkey anti-rabbit IgG (H+L)	Invitrogen	A-10040	1:300
Alexa Fluor 568 donkey anti-rabbit IgG (H+L)	Invitrogen	A-10042	1:300
Alexa Fluor 488 donkey anti-mouse IgG (H+L)	Invitrogen	A-21202	1:300
Alexa Fluor 647 donkey anti-chicken IgY (H+L)	Jackson ImmunoResearch	703-605-155	1:300
Alexa Fluor 546 goat anti-chicken IgY (H+L)	Invitrogen	A-11040	1:250

### Transmission electron microscopy

Ovary grafts collected at 2 weeks post KCT surgery were processed for transmission electron microscopy by the University of Wisconsin Electron Microscopy Service. Briefly, samples were immersion fixed for 2 hours in 2.5% glutaraldehyde, 2% PFA buffered in 0.1M sodium phosphate buffer (PB) at room temperature (RT). After rinsing, samples were post-fixed in 1% osmium tetroxide, 1% potassium ferrocyanide in 0.1M PB for 1 hour at RT, rinsed, and then stained in saturated aqueous uranyl acetate for 2 hours at RT. Dehydration was performed at RT in a graded ethanol series and then transitioned in propylene oxide (PO). Fully dehydrated samples were then infiltrated in increasing concentrations of PolyBed 812 (Polysciences Inc. Warrington, PA) and PO mixtures. Embedding and polymerization took place in fresh PolyBed 812 for 48 hours at 60°C. Semi-thin sections (1 mm) were first stained with methylene blue/Azure II for light microscopic inspection. The samples were then sectioned on a Leica EM UC6 ultramicrotome at 90nm. The sections were collected on Cu, 300 mesh thin-bar (EMS Hatfield, PA), and post-stained in uranyl acetate and lead citrate. The sectioned samples were viewed at 80kV on a Philips CM120 transmission electron microscope, equipped with MegaView III camera (Olympus Soft Imaging System Lakewood, CO).

### Statistical analyses

Statistical evaluation of superovulation, IVF, breeding study, follicle quantification, and laminin intensity quantification results between groups were carried out using a two-tailed t-test assuming unequal variances. Results were considered statistically different if p-values were ≤ 0.05. Results of p < 0.2 are also reported. Gene expression value analysis was conducted as described in the figure legend.

## Supporting information

S1 Fig Examples of abnormal follicle criteria. (A-D) Follicles in 10-day, 2-week and 3-week KCT control grafts show normal morphology. (E-H) *Irx3/5* DKO follicles in 10-day, 2-week and 3-week KCT grafts exhibit abnormal morphology (follicles outlined with yellow dashed line), such as mis-shaped granulosa cells (E, F), wisp-like oocyte cytoplasm (G,H), asymmetric accumulation of granulosa cells relative to a central oocyte (G), and increased distances between granulosa cells and/or between granulosa cells and oocytes (G, H). Scale bars: 5 μm.
(TIFF)

S2 Fig *Irx3/5* hypomorph oocytes have a higher fragmentation rate, but healthy oocytes from mutant and control groups subjected to IVF develop to 2-cell embryos at a similar rate. (A) The fragmented oocyte rate was calculated by dividing the number of fragmented oocytes by the total number of oocytes for each animal after superovulation. Then, each animal was normalized to the average of the control animals in each experiment. (B) Only healthy (non-fragmented) oocytes from both groups were used for IVF experiments. The proportion of fertilized oocytes that progressed to the 2-cell embryo stage was calculated by dividing the 2-cell embryo number by the number of oocytes subjected to fertilization for each animal. This proportion was then normalized to the averaged control proportion within each experiment. Data in both graphs represent mean ± SEM. Statistics: two-sample t-test; #: p<0.2. Control, black bars; *Irx3/5* hypomorph, hatched bars. (C-D) Scatter plots representing the raw data for oocyte numbers and 2-cell embryo numbers after superovulation and IVF. Bars in both graphs represent mean ± SEM. Control, black dots; *Irx3/5* hypomorph, red squares.
(TIFF)

S3 Fig *Irx3* and *Irx5* transcripts are female specific and peak around the time of birth. (A) Real-time qPCR results for *Irx3* in wild-type male and female gonads from E12.0 to adulthood. (B) Real-time qPCR results for *Irx5* in wild-type male and female gonads from E12.0 to adulthood. Data represents the mean ± SEM of three biological replicates performed in triplicate at each time point. Fold change was calculated relative to transcript levels of the female gonad at E12.0. Statistics: two-way ANOVA with Bonferroni post-hoc test, a: statistical significance between time point and E12.0; b: statistical significance between sex at each time point (marked as significant if at least p < 0.01).
(TIFF)

S4 Fig *Irx3* and *Irx5* express in GATA4-positive somatic cells during germline nest stage. *Irx3-LacZ* and *Irx5-EGFP* ovaries were examined at E15.5. (A) Immunofluorescence of *Irx3* (*Irx3-LacZ*, green) with somatic cell marker GATA4 (red, nucleus). (B) Immunofluorescence of *Irx5* (*Irx5-EGFP*, green) with GATA4 (red, nucleus). (A’ and B’) Enlarged views of the area of interest that is white boxed in A and B, respectively. Asterisk (*): germline nest. Scale bars: 50 μm.
(TIFF)

S5 Fig Granulosa cells of *Irx3/5* DKO follicles do not transdifferentiate into Sertoli cells. Ovarian follicles of 2-week KCT grafts examined for FOXL2 (A, B, red) and AMH (C, D, red) in control (A, C) and *Irx3/5* DKO (B, D) follicles. (E-G) Immunofluorescence of a Sertoli cell marker, SOX9 (red) in control (E) and *Irx3/5 DKO* (F) follicles. E16.6 testis (G) is used as a positive control for SOX9. White circle: outline of follicle. Scale bars: 50 μm.
(TIFF)

S6 Fig *Laminin* and *Gja1* transcript levels are not different between control and *Irx3/5* DKO ovary samples at E13.5 and 7d KCT. Transcript levels are reported from RNA-Seq data from E13.5 and 7d KCT time points. E13.5 ovaries of *Irx3/5* DKO (n = 6) and WT control (n = 6) mice were processed for RNA extraction. Seven-day KCT grafts of *Irx3/5* DKO and heterozygous control (*Irx3* ^*-*^*Irx5*^*EGFP*^/*Irx3*^*+*^*Irx5*^*+*^) ovaries were dissociated into single cell suspension and sorted using fluorescence activated cell sorting (FACS). GFP positive cells were collected and processed for RNA extraction. RNA from graft samples were pooled and 3 biological samples were used for control and *Irx3/5* DKO groups. Preliminary RNA-Seq results show comparable levels of both *Laminin* (A) and *Gja1* (B) in control (black columns) and *Irx3/5* DKO (blue columns) ovary samples. Data represents mean ± SD. Statistics: two-sample t-test.
(TIFF)

S7 Fig *Irx3/5* DKO follicle exhibits ectopic GJA1 expression. Primary follicles of 10-day KCT grafts are examined for GJA1 expression. (A) Double immunofluorescence of GJA1 (red) and VASA (green, germ cell marker) in control follicles. (B, C) Immunofluorescence of GJA1 (red) with VASA (green) and GATA4 (green, somatic cells marker) in *Irx3/5* DKO follicles (arrow: expected location of GJA1 between granulosa cells; arrowhead: ectopic GJA1 expression between granulosa cells and the oocyte).
(TIFF)

S8 Fig *Irx3/5* DKO follicles did not show increased cell apoptosis activity. Two-week and 3-week KCT grafts of *Irx3/5* DKO and wild-type control ovaries were stained for cleaved caspase 3 (red) for cell apoptosis activity. Laminin (green) was used to define follicle boundaries. White arrows indicate examples of follicles. Few follicles expressed cleaved caspase three, examples are highlighted in panels A and B. The white dashed circles in C and D outline duct-like structures (not follicles) in grafts that are positive for both cleaved caspase 3 and laminin. Scale bars: 100 μm.
(TIFF)

S1 Table Summary table of the IRX3 expression profile during follicle formation and maturation. (DOCX)

S2 Table Summary table of quantified comparisons of basement membrane features from TEM images of Irx3/5 DKO vs wild type controls. (DOCX)

S1 Text Supplemental methods. (DOCX)
