# Supplementary figures and images for: Dynamic expression patterns of Irx3 and Irx5 during germline nest breakdown and primordial follicle formation promote follicle survival in mouse ovaries
Source: PLoS Genet. 2018 Aug 2;14(8):e1007488. doi: 10.1371/journal.pgen.1007488 (PMC6071956; doi:10.1371/journal.pgen.1007488)

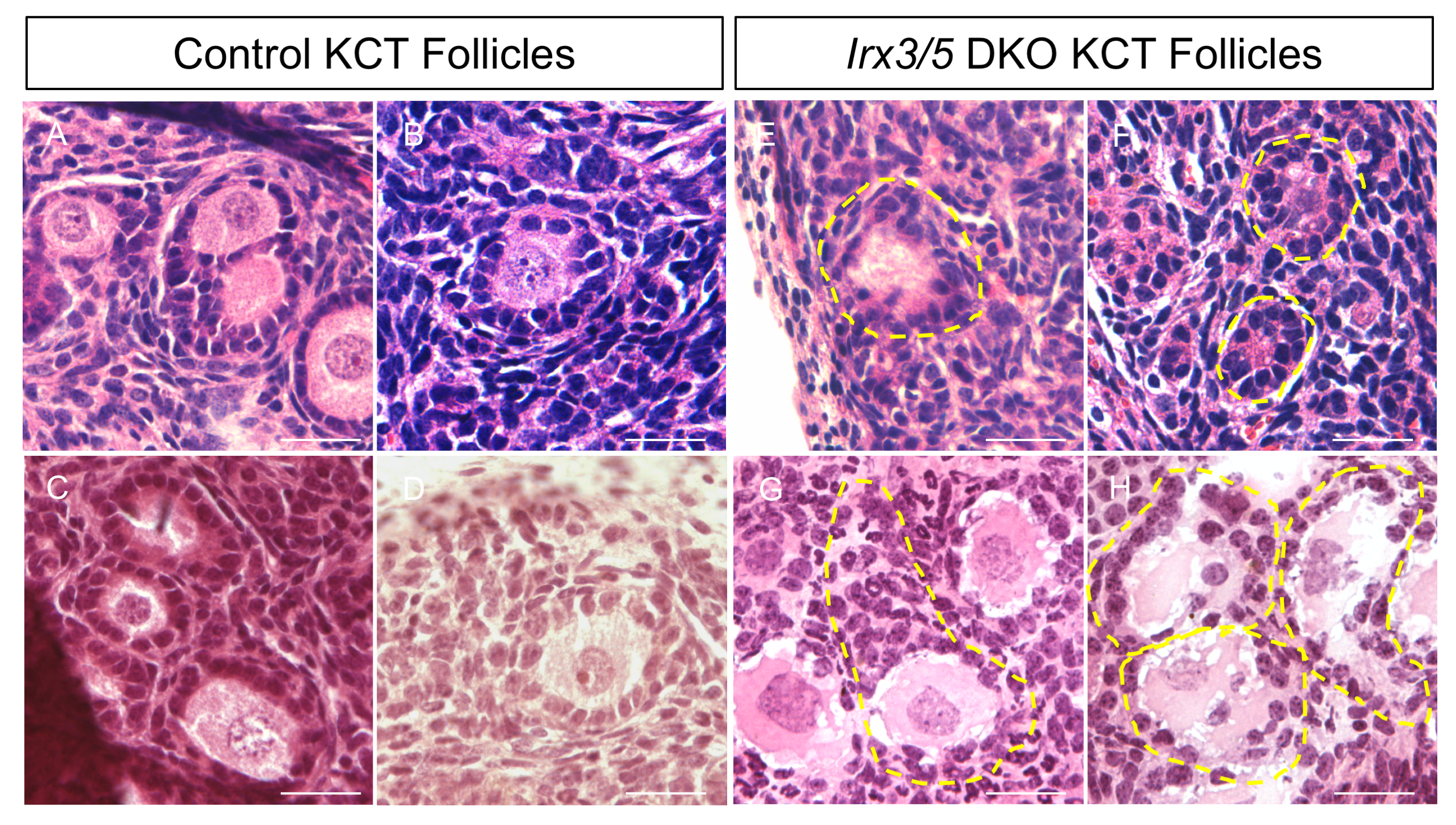

Supplement: S1 Fig — (A-D) Follicles in 10-day, 2-week and 3-week KCT control grafts show normal morphology. (E-H) Irx3/5 DKO follicles in 10-day, 2-week and 3-week KCT grafts exhibit abnormal morphology (follicles outlined with yellow dashed line), such as mis-shaped granulosa cells (E, F), wisp-like oocyte cytoplasm (G,H), asymmetric accumulation of granulosa cells relative to a central oocyte (G), and increased distances between granulosa cells and/or between granulosa cells and oocytes (G, H). Scale bars: 5 μm. (TIFF) [file pgen.1007488.s001.tiff]

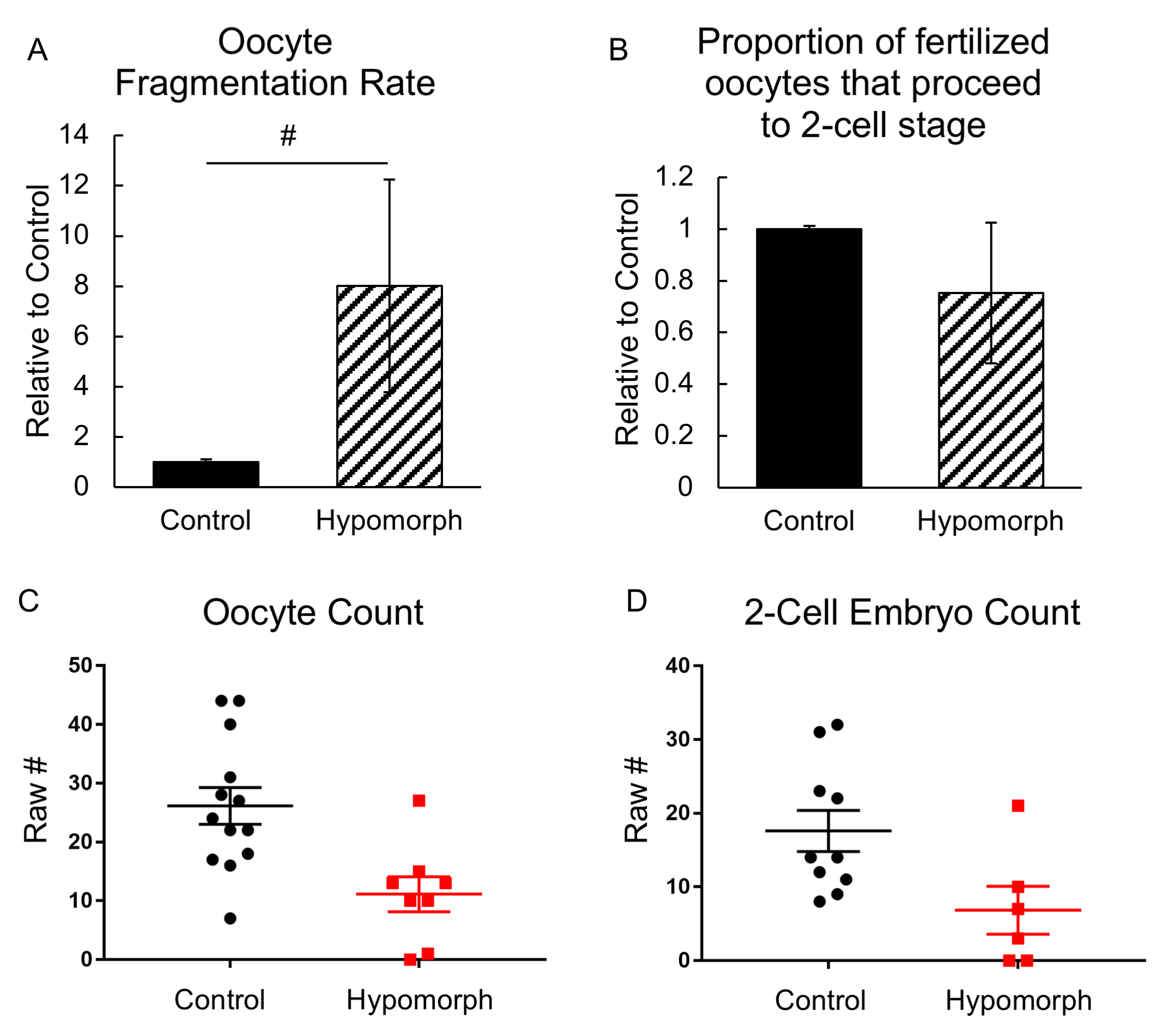

Supplement: S2 Fig — (A) The fragmented oocyte rate was calculated by dividing the number of fragmented oocytes by the total number of oocytes for each animal after superovulation. Then, each animal was normalized to the average of the control animals in each experiment. (B) Only healthy (non-fragmented) oocytes from both groups were used for IVF experiments. The proportion of fertilized oocytes that progressed to the 2-cell embryo stage was calculated by dividing the 2-cell embryo number by the number of oocytes subjected to fertilization for each animal. This proportion was then normalized to the averaged control proportion within each experiment. Data in both graphs represent mean ± SEM. Statistics: two-sample t-test; #: p<0.2. Control, black bars; Irx3/5 hypomorph, hatched bars. (C-D) Scatter plots representing the raw data for oocyte numbers and 2-cell embryo numbers after superovulation and IVF. Bars in both graphs represent mean ± SEM. Control, black dots; Irx3/5 hypomorph, red squares. (TIFF) [file pgen.1007488.s002.tiff]

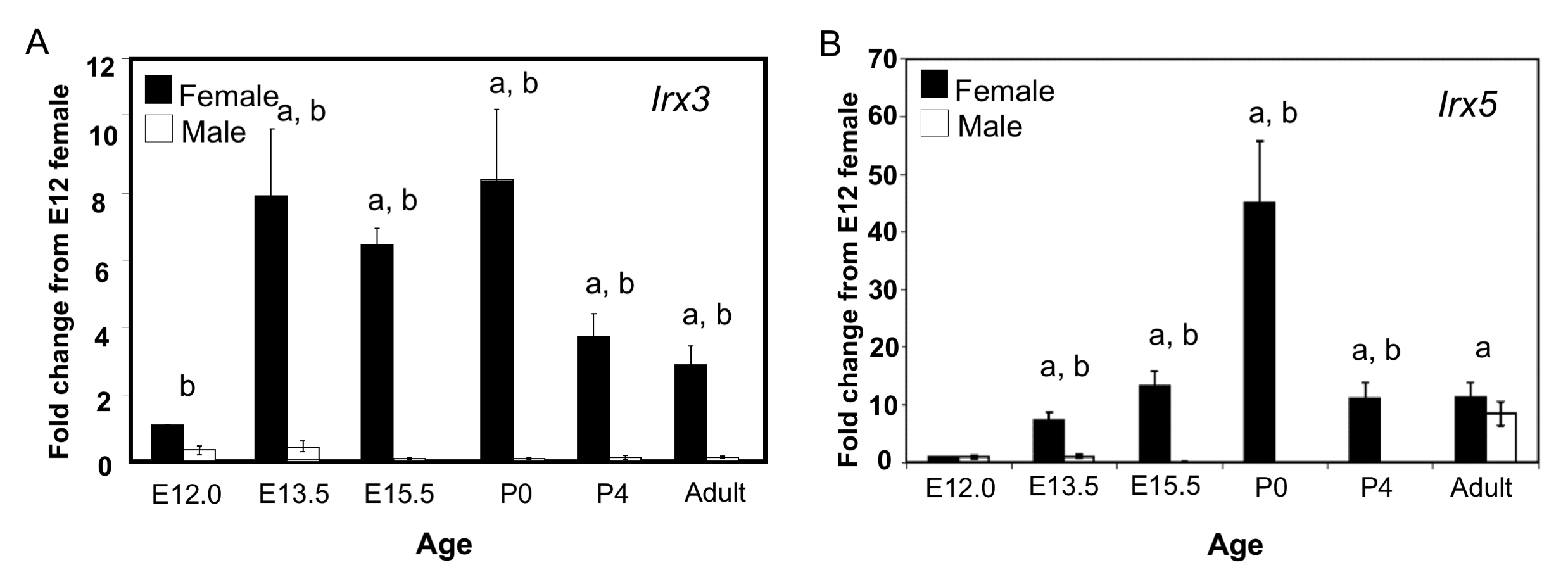

Supplement: S3 Fig — (A) Real-time qPCR results for Irx3 in wild-type male and female gonads from E12.0 to adulthood. (B) Real-time qPCR results for Irx5 in wild-type male and female gonads from E12.0 to adulthood. Data represents the mean ± SEM of three biological replicates performed in triplicate at each time point. Fold change was calculated relative to transcript levels of the female gonad at E12.0. Statistics: two-way ANOVA with Bonferroni post-hoc test, a: statistical significance between time point and E12.0; b: statistical significance between sex at each time point (marked as significant if at least p < 0.01). (TIFF) [file pgen.1007488.s003.tiff]

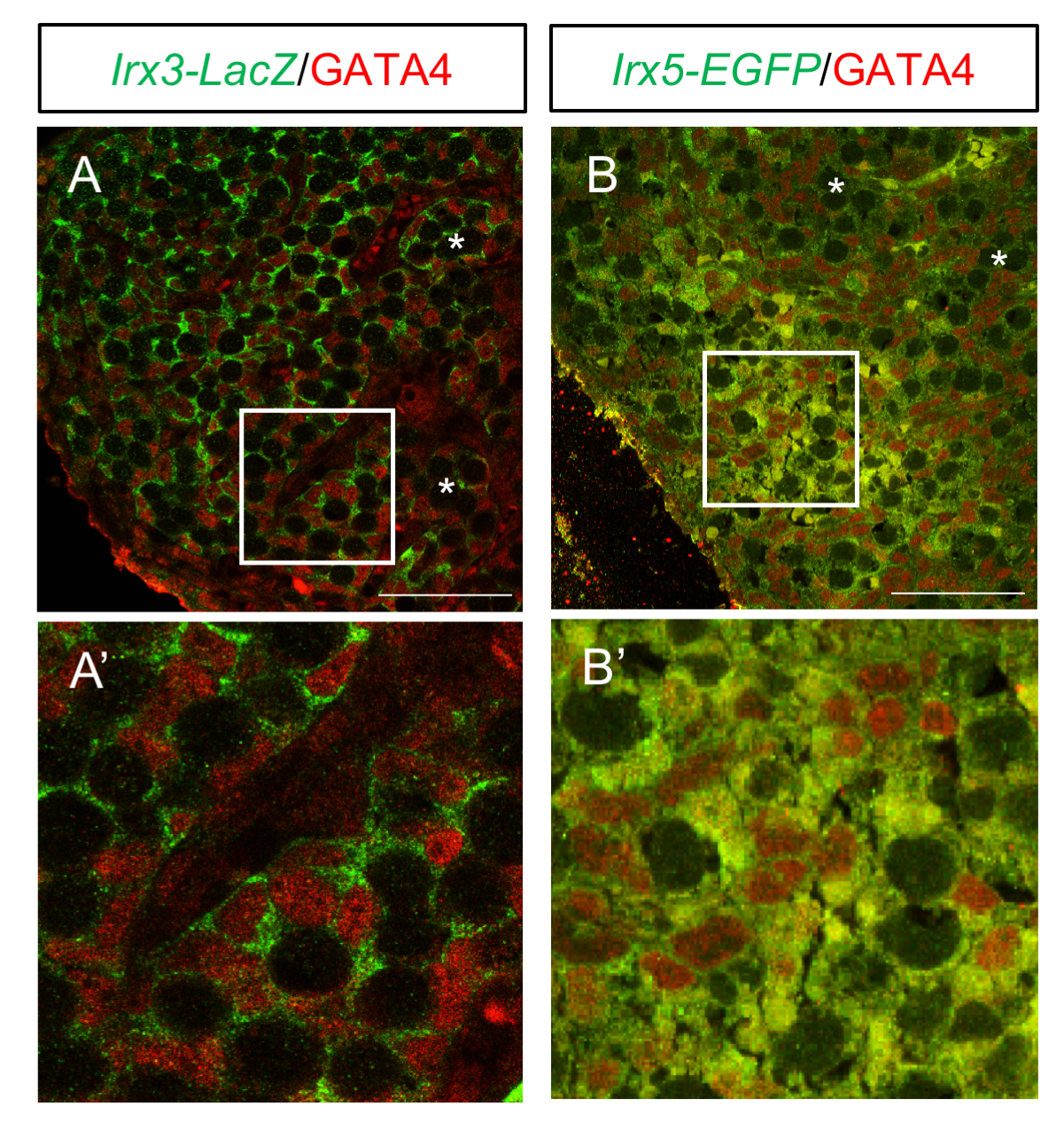

Supplement: S4 Fig — Irx3-LacZ and Irx5-EGFP ovaries were examined at E15.5. (A) Immunofluorescence of Irx3 (Irx3-LacZ, green) with somatic cell marker GATA4 (red, nucleus). (B) Immunofluorescence of Irx5 (Irx5-EGFP, green) with GATA4 (red, nucleus). (A’ and B’) Enlarged views of the area of interest that is white boxed in A and B, respectively. Asterisk (*): germline nest. Scale bars: 50 μm. (TIFF) [file pgen.1007488.s004.tiff]

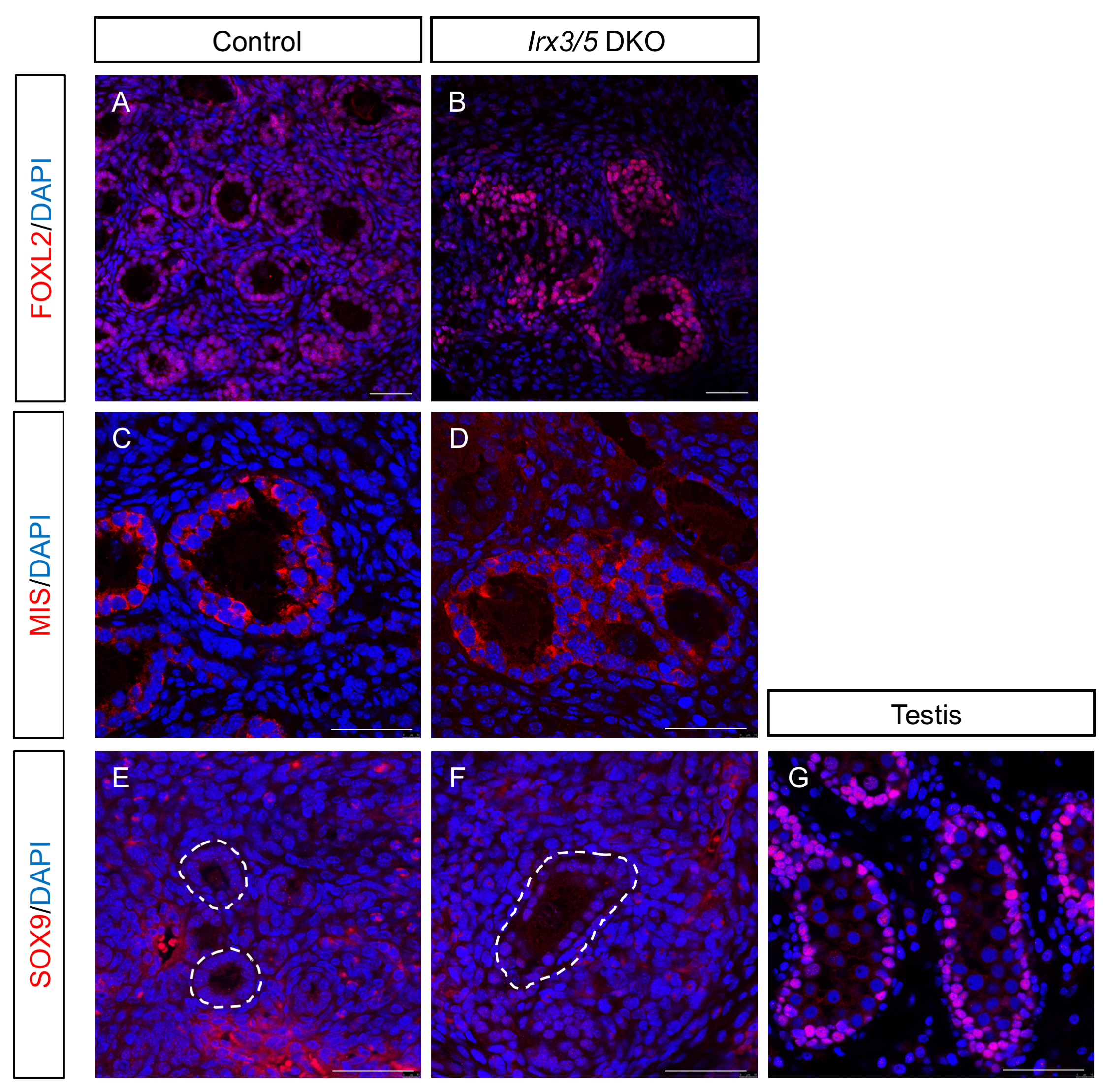

Supplement: S5 Fig — Ovarian follicles of 2-week KCT grafts examined for FOXL2 (A, B, red) and AMH (C, D, red) in control (A, C) and Irx3/5 DKO (B, D) follicles. (E-G) Immunofluorescence of a Sertoli cell marker, SOX9 (red) in control (E) and Irx3/5 DKO (F) follicles. E16.6 testis (G) is used as a positive control for SOX9. White circle: outline of follicle. Scale bars: 50 μm. (TIFF) [file pgen.1007488.s005.tiff]

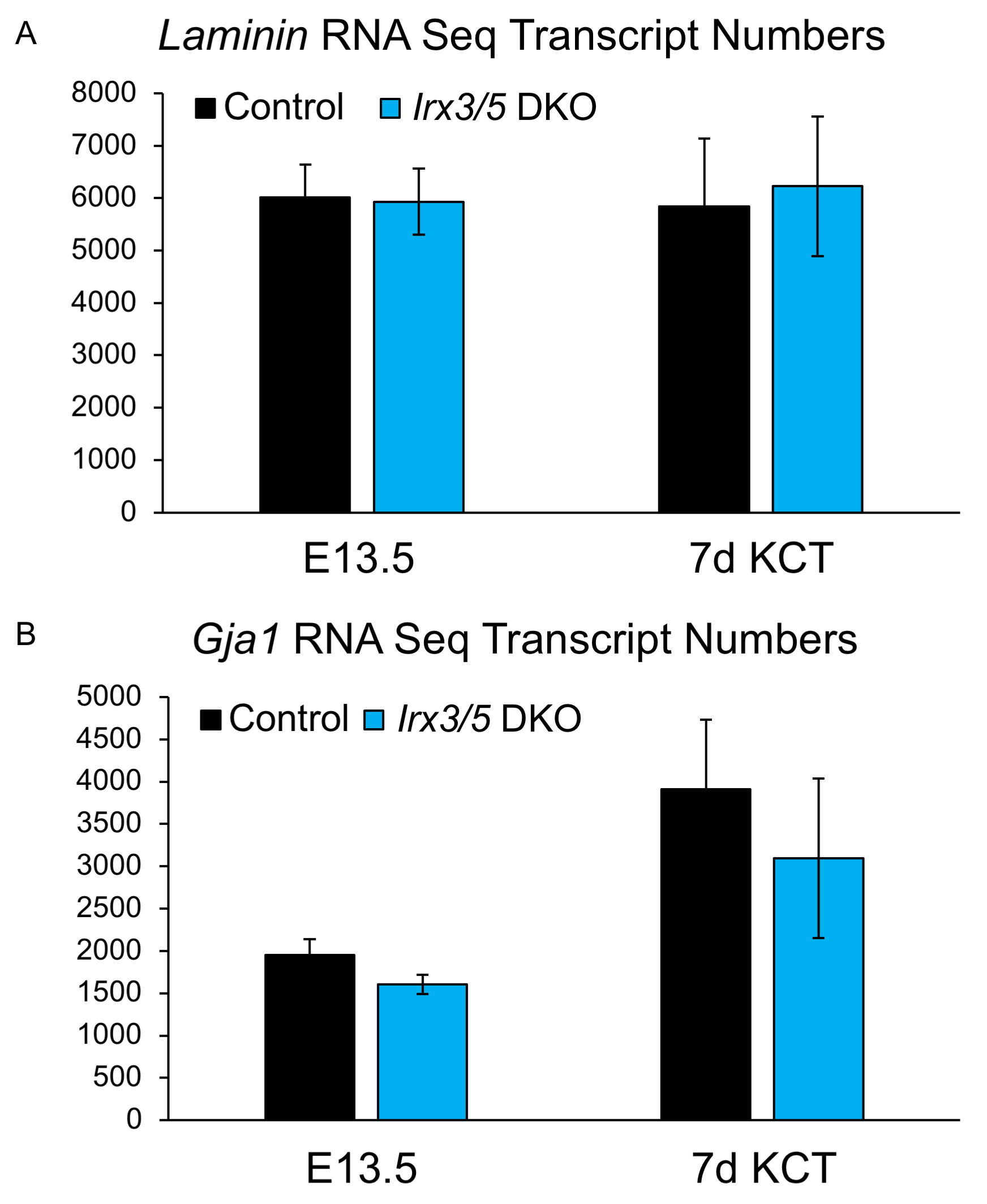

Supplement: S6 Fig — Transcript levels are reported from RNA-Seq data from E13.5 and 7d KCT time points. E13.5 ovaries of Irx3/5 DKO (n = 6) and WT control (n = 6) mice were processed for RNA extraction. Seven-day KCT grafts of Irx3/5 DKO and heterozygous control (Irx3 -Irx5EGFP/Irx3+Irx5+) ovaries were dissociated into single cell suspension and sorted using fluorescence activated cell sorting (FACS). GFP positive cells were collected and processed for RNA extraction. RNA from graft samples were pooled and 3 biological samples were used for control and Irx3/5 DKO groups. Preliminary RNA-Seq results show comparable levels of both Laminin (A) and Gja1 (B) in control (black columns) and Irx3/5 DKO (blue columns) ovary samples. Data represents mean ± SD. Statistics: two-sample t-test. (TIFF) [file pgen.1007488.s006.tiff]

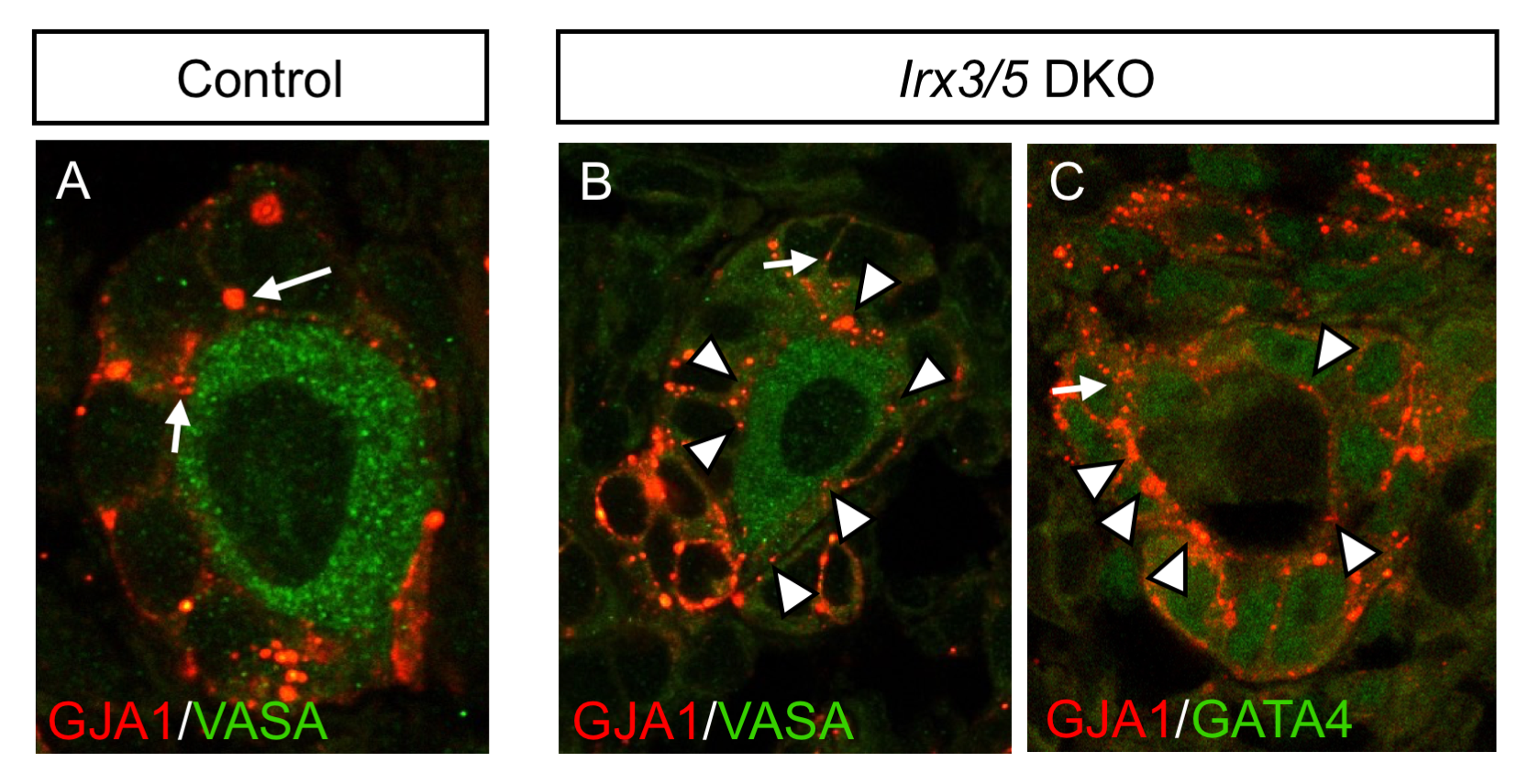

Supplement: S7 Fig — Primary follicles of 10-day KCT grafts are examined for GJA1 expression. (A) Double immunofluorescence of GJA1 (red) and VASA (green, germ cell marker) in control follicles. (B, C) Immunofluorescence of GJA1 (red) with VASA (green) and GATA4 (green, somatic cells marker) in Irx3/5 DKO follicles (arrow: expected location of GJA1 between granulosa cells; arrowhead: ectopic GJA1 expression between granulosa cells and the oocyte). (TIFF) [file pgen.1007488.s007.tiff]

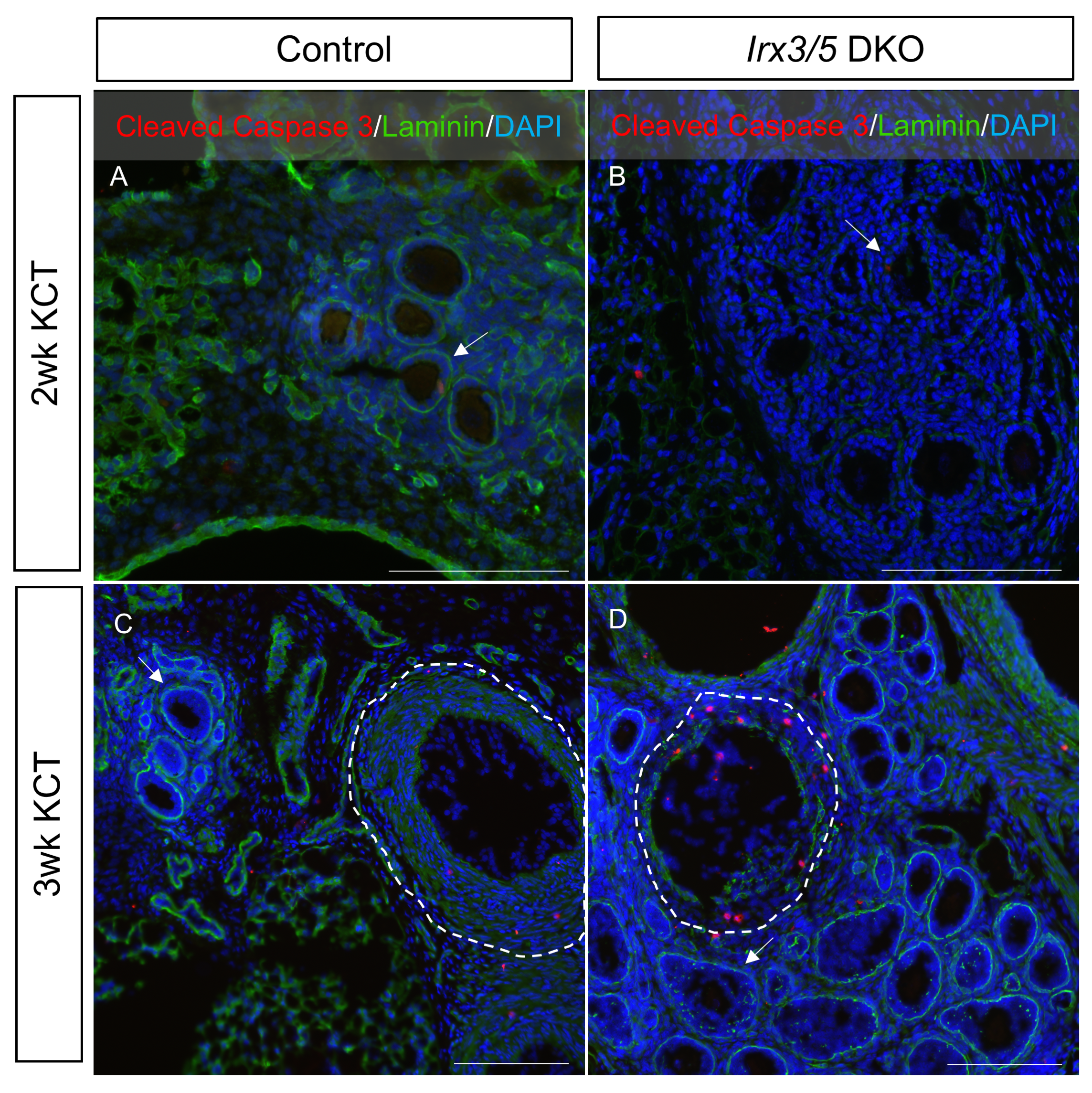

Supplement: S8 Fig — Two-week and 3-week KCT grafts of Irx3/5 DKO and wild-type control ovaries were stained for cleaved caspase 3 (red) for cell apoptosis activity. Laminin (green) was used to define follicle boundaries. White arrows indicate examples of follicles. Few follicles expressed cleaved caspase three, examples are highlighted in panels A and B. The white dashed circles in C and D outline duct-like structures (not follicles) in grafts that are positive for both cleaved caspase 3 and laminin. Scale bars: 100 μm. (TIFF) [file pgen.1007488.s008.tiff]
