# Supplementary material for: Dynamic expression patterns of Irx3 and Irx5 during germline nest breakdown and primordial follicle formation promote follicle survival in mouse ovaries
Source: PLoS Genet. 2018 Aug 2;14(8):e1007488. doi: 10.1371/journal.pgen.1007488 (PMC6071956; doi:10.1371/journal.pgen.1007488)
Supplement: S1 Table — (DOCX) [file pgen.1007488.s009.docx]

| Supplemental Table 1 | | | |  |  |  |  |  |  |
| --- | --- | --- | --- | --- | --- | --- | --- | --- | --- |
| IRX3 Expression Profile During Follicle Formation and Maturation | | **Germline Cyst** | | **Primordial Follicle** | | **Primary Follicle** | | **Older Follicles** | |
|  |  | Nucleus | Cytoplasm | Nucleus | Cytoplasm | Nucleus | Cytoplasm | Nucleus | Cytoplasm |
| Early Stage | Somatic Cells | + | - | + | - | - | - | - | - |
|  | Germ cells | - | - | + | + | + | + | + | + |
| Late Stage | Somatic Cells | + | - | - | - |  |  |  |  |
|  | Germ Cells | + | + | + | + |  |  |  |  |
