# Supplementary material for: Dynamic expression patterns of Irx3 and Irx5 during germline nest breakdown and primordial follicle formation promote follicle survival in mouse ovaries
Source: PLoS Genet. 2018 Aug 2;14(8):e1007488. doi: 10.1371/journal.pgen.1007488 (PMC6071956; doi:10.1371/journal.pgen.1007488)
Supplement: S2 Table — (DOCX) [file pgen.1007488.s010.docx]

| Supplemental Table 2 | | |  |  |  |  |
| --- | --- | --- | --- | --- | --- | --- |
|  | BM length evaluated | Average BM thickness | | % Length thickened BM* | Incidence double BM | Incidence looped BM |
| Wild type | 31.368μm | 0.054 +/- 0.013μm | | 19.8% | 0 | 0 |
| Irx3/5 DKO | 45.839μm | 0.056 +/- 0.0089μm | | 34.9% | 2 | 4 |
| BM = basement membrane | |  | |  |  |  |
| * cutoff = average + one SD BM thickness | | | |  |  |  |
