# Supplementary material for: Dynamic expression patterns of Irx3 and Irx5 during germline nest breakdown and primordial follicle formation promote follicle survival in mouse ovaries
Source: PLoS Genet. 2018 Aug 2;14(8):e1007488. doi: 10.1371/journal.pgen.1007488 (PMC6071956; doi:10.1371/journal.pgen.1007488)
Supplement: S1 Text — (DOCX) [file pgen.1007488.s011.docx]

RNA Extraction and Quantitative Real-Time PCR (qPCR)

RNA was prepared from pooled testes or ovaries collected from E12.0, E13.0, E15.5, P0, P4 and 6-week- to 7-week-old mice using RNeasy Micro Kit (Qiagen). All real-time PCR assays were carried out using the SYBR Green I Kit (Applied Biosystems) as previously described in [28]. The expression level analysis and gene specific primer sequences were previously described in [23] and [29]. RNA isolated from E13.5 ovaries and 7d KCT ovary grafts was submitted to UW Biotech Center for library preparation with NuGen Ovation SoLo kit and sequenced on Illumina HiSeq2500.
